# Ten questions about terminology for children with unexplained language problems

**DOI:** 10.1111/1460-6984.12101

**Published:** 2014-08-20

**Authors:** D V M Bishop

**Affiliations:** Department of Experimental Psychology, University of OxfordOxford, UK

**Keywords:** diagnosis, DSM-5, labels, terminology, specific language impairment

## Abstract

**Background:**

In domains other than language, there is fairly consistent diagnostic terminology to refer to children's developmental difficulties. For instance, the terms ‘dyslexia’, ‘attention deficit hyperactivity disorder’ and ‘autistic spectrum disorder’ are used for difficulties with reading, attention or social cognition, respectively. There is no agreed label, however, for children with unexplained language problems.

**Aims:**

To consider whether we need labels for unexplained language problems in children, and if so, what terminology is appropriate.

**Main Contribution:**

There are both advantages and disadvantages to labels, but they are important to ensure children receive services, and to increase our knowledge of the nature and causes of such problems. A survey of labels in current use found 132 different terms, 33 of which had 600 or more returns on Google Scholar between 1994 and 2013. Many of these labels were too general to be useful. Of the remainder, the term ‘specific language impairment’ was the most commonly used.

**Conclusions:**

The current mayhem in diagnostic labels is unsustainable; it causes confusion and impedes research progress and access to appropriate services. We need to achieve consensus on diagnostic criteria and terminology. The DSM-5 term ‘language disorder’ is problematic because it identifies too wide a range of conditions on an internet search. One solution is to retain specific language impairment, with the understanding that ‘specific’ means idiopathic (i.e., of unknown origin) rather than implying there are no other problems beyond language. Other options are the terms ‘primary language impairment’, ‘developmental language disorder’ or ‘language learning impairment’.

**What this paper adds?**

This paper aims to open up discussion about the use of different labels that have been used to refer to children's unexplained language impairments. It notes the wide range of terminology that has been applied and the confusion that results, and links this to debates about the appropriate criteria that are used to identify children in need of intervention. A range of diagnostic terms are evaluated in terms of their advantages and disadvantages.

## Introduction

Consider the case of 8-year-old George. He was rather late to start talking, and he did not speak in sentences until he was 4 years old. In other regards he developed normally: he is a healthy child and a hearing check found no problems. He attends mainstream school, but he struggles with reading, and has a weak vocabulary for his age. He does not always remember what his teacher says to him, and his confidence, never good, has been dented further by other boys teasing him for not understanding the punch line to a joke. George is having some extra help with his reading in a small group, but he hates being singled out and made to feel different from others. He is beginning to be reluctant to go to school, except on days when he has art lessons, which he loves. His parents, concerned to see him so miserable, have arranged a private assessment with a psychologist, who diagnoses specific language impairment (SLI) and dyslexia. She explains that George has a nonverbal IQ of 95, within normal limits, but his vocabulary and comprehension levels are lower, with scaled score equivalents of 80, and his reading ability is at a 6-year-old level. The parents look for information on the internet and learn that SLI is thought to be a strongly genetic disorder that impairs language development. When, however, they talk to the head teacher about the assessment, he is not impressed. He thinks that it is unhelpful to apply a diagnostic label to George. All children vary in their language abilities, he explains, and the best approach will be to continue to support George with extra help in the classroom. He offers to ask the speech and language therapist for her opinion, as she is good at working with teachers to find the best way to help children with speech, language and communication needs (SLCN). The head teacher explains that there have been growing concerns that too many children are being identified with special educational needs (SEN), which just creates stigma and low expectations.

This vignette illustrates a number of tensions that surround the identification and labelling of children whose language development is falling behind their peer group for no obvious reason. There is polarization between two extremes: those who treat identification of children's language problems as akin to medical diagnosis, and those who adopt a normative approach, which eschews diagnostic labels as invalid and inappropriate. Among those who use labels, there is no agreement as to what is appropriate. In reviewing background literature, I shall use the term ‘specific language impairment’ (SLI) when referring to studies that have used this term, but provisionally will otherwise talk of ‘unexplained language problems’. The vexed issue of what terminology should be adopted will emerge in the course of this article.

## 1. Should we be concerned about children's language problems?

Should we just let children develop at their own pace rather than worrying about those who progress more slowly for no apparent reason? On this point, I suspect there will be agreement between most professionals, regardless of which discipline they come from. The evidence is stark: children whose language lags well behind their peer group are at increased risk of academic failure (Durkin *et al*. 2012, Johnson *et al*. 2010), behavioural and psychiatric problems (Conti-Ramsden *et al*. 2013, Snowling *et al*. 2006), unemployment and economic disadvantage (Parsons *et al*. 2011), and social impairment (Clegg *et al*. 2005).

Age, however, is critical. Late-talking in toddlers is not necessarily predictive of future problems, provided language comprehension is adequate, there is no family history of language or literacy problems, and other aspects of development are proceeding on course (Lyytinen *et al*. 2005, Zambrana *et al*. 2014). Many late-talkers catch up with their peer group after a slow start, and do not have significant difficulties later on (Reilly *et al*. 2010). But for children whose language deficits persist into school age, the outlook is bleaker (Conti-Ramsden and Durkin 2008, Stothard *et al*. 1998, Tomblin *et al*. 2003), prompting concern about whether we can effectively intervene to prevent a downward spiral of negative consequences.

## 2. Should we abandon diagnostic labels?

In many educational contexts, there is resistance to giving children diagnostic labels. The approach is educational rather than medical, with the goal being to identify children who will benefit from help by identifying the specific kinds of need on an individual basis. The more generic term ‘special educational needs’ (SEN) is used to determine who gains access to special educational provision; this would encompass children with serious communication difficulties alongside those with other disabilities affecting education. ‘Speech, language and communication needs’ (SLCN) is used as a nonspecific term, i.e., it covers a range of children including those with English as an additional language, stuttering, or speech/language problems due to hearing loss or physical causes, as well as those with unexplained language problems. Within the UK educational system, diagnostic labels are not widely adopted, and the Diagnostic and Statistical Manual (DSM-5) classification system of the American Psychiatric Association (2013) and International Classification of Diseases (ICD-10) of the World Health Organization (1992) are largely ignored.

For some, labelling is seen as irrelevant, whereas for others, it is explicitly rejected as having more negative than positive consequences. Some of the disadvantages of diagnostic labels are summarized in the first column of table1, which draws heavily on arguments advanced by Lauchlan and Boyle (2007).

**Table 1:** Pros and cons of diagnostic labels

Negative consequences	Positive consequences
Focus on what is wrong with the child; may ignore aspects of environment; localize problem in the child	Provides an explanation and legitimacy
Parents take no responsibility	Removes blame from parents
Child feels failure inevitable, stops trying	Removes blame from child
Excuse for what is really consequence of bad teaching	Removes blame from teachers
Leads to stigmatization, social disadvantage and exclusion	Promotes understanding and awareness of particular difficulties; legal protection against discrimination; can give sense of belonging: support groups; allows for group action; can lead to emphasis on positive attributes
Resources denied to those who do not meet specific diagnostic criteria; cynical use of labels to get extra funds	Leads to access to resources; in some countries may not be able to access these without a diagnostic label
Focus on label rather than assessment of child's specific needs; tendency to stereotype; generalizations may obscure important differences	Recognize common patterns across children with similar difficulties
Child may do better with skilled teaching and not need/ benefit from other intervention	Child can receive targeted intervention
Same label used with different meanings leads to confusion	Facilitates communication among professionals
Undue reliance on unreliable criteria, especially IQ	Objective criteria from formal assessment identify problems that might otherwise get missed
Medicalization of non-medical disorders; social problems attributed to medical causes	Recognition of biological as well as social causes of difficulties
Planning in terms of numbers with difficulties, rather than making changes that benefit all children	Need to know how many children affected, for planning resources and documenting progress
Groups studied by researchers are artificial and findings may not generalize to most children	Researchers need to generalize across groups; labels allow for continuity across research

Avoidance of labels may seem an admirably pragmatic approach which avoids potential stigmatization. It also avoids the unfairness that can ensue if educational support is restricted to those who meet arbitrary cut-offs, such as the discrepancy criteria sometimes used to identify children with specific learning disabilities (Fletcher 1992). It does, however, have some serious limitations. First, in avoiding medicalization of children's difficulties, we may swing too far in the other direction, denying any role of biological risk factors in causing problems. The net result can be a culture of blaming either the parents or the teachers when children fail to achieve. A more balanced approach recognizes that children vary in their biological as well as their social backgrounds, and educational approaches need to be optimal for each individual, without introducing notions of inadequacy or blame.

Second, without diagnostic categories, it becomes easy for educational and governmental agencies to minimize children's difficulties, especially if they are attributed to poor schooling. With no clear criteria for deciding who needs extra help, it is all too easy to remove support. Consider, for instance, a government report issued in 2010 that argued that there was massive over-identification of children with SEN (Office for Standards in Education, Children's Services and Skills 2010). The authors of this report took the view that a primary reason for children's educational failures was inadequate teaching, and that schools were using the terminology of SEN to disguise their limitations and imply that the reason for failure lay in the child rather than in poor teaching. If there are no agreed criteria of what constitutes a significant language problem, then it is impossible for anyone to provide evidence either for or against this statement—it is simply a matter of opinion as to who merits special help. If we had clear and objective criteria, we could then gather evidence to determine which children actually benefit from support and services.

This leads us to the third limitation of the ‘no labels’ approach, namely that it hampers research. In order to find out more about the nature and causes of language problems, and to discover which interventions are effective, we need to study groups of children. We can only do that if we can agree who is to be in the group, and hence we need to agree on diagnostic criteria. To date, researchers have had notable successes in finding out about the linguistic difficulties, correlates, outcomes and causes of SLI, despite the fuzziness and heterogeneity of this diagnostic category. For example, we have been able to identify specific deficits that might help account for language difficulties (Conti-Ramsden *et al*. 2001), to evaluate efficacy of intervention (Washington *et al*. 2011), to give parents a prognosis (Whitehouse *et al*. 2009a), and to identify genetic risk factors (Bishop *et al*. 1995): Our knowledge is far from perfect, but it would be non-existent if we had not been able to identify groups for study. None of this would be possible using a global category such as SLCN, which may be workable for certain administrative purposes, but is too broad for research contexts. It is sometimes argued that in identifying children with SLI, we are assuming they are all the same. That is wrong: they will differ in various ways, but the point is that we can identify clusters of children who share some key characteristics. In clinical contexts, we need to beware of stereotyping and assuming all children are the same, but if we treat each child as unique, we can never generalize and learn from our experiences.

Arguments about labelling are not confined to the field of language impairment, or even to neurodevelopmental disorders. In his critique of DSM-5, *Saving Normal*, Frances (2013) noted the societal significance of labels in psychiatry. He was particularly concerned about the expansion of diagnostic categories in DSM-5, whereby normal variations in behaviour were being treated as diseases, so that a very high proportion of the population would qualify for a diagnosis. Nevertheless, Frances was careful to stress that he was not opposed to diagnostic labels—quite the contrary. He noted that in situations where resources are limited—which is almost always—budgets are a zero-sum game: if you do not have a diagnosis, then nobody will pay for your treatment.

Overall, Frances's conclusions have broad applicability to the case of children's language problems. There is a necessity for diagnostic labels if we are to advance our understanding of why some children have language problems, and identify those who might benefit from intervention. However, there is considerable potential for unintended consequences from labelling, and we need to think carefully about what kind of labels we use and whether we can take steps to mitigate the negative impacts that can arise from their use.

## 3. Is a medical model appropriate for unexplained language problems in children?

Does use of diagnostic labels ‘medicalize’ children's difficulties inappropriately? After all, language difficulties are quite different from a condition such as Down syndrome, where there is a known aetiology (an extra copy of chromosome 21), leading to a distinctive cluster of physical and cognitive characteristics. Labels may give the impression that they offer explanations for children's difficulties, especially when they are medical-sounding, like ‘dyslexia’ or ‘Asperger syndrome’, but in fact these are behaviourally defined conditions, and the labels are really no more than shorthand descriptions of a cognitive profile. The drawback of medical labels is that they can lead to what Hyman (2010) has termed ‘reification’: the assumption that our labels are defining ‘natural kinds’.

SLI is not a distinct syndrome. There is evidence for genetic variants that increase the risk of language impairment (Newbury *et al*. 2011), but individual genes typically have very small effects, and, importantly, the genetic variants associated with increased risk are common in the general population. Rare mutations that cause major language problems are the exception rather than the rule (Graham and Fisher 2013). SLI is best conceptualized as a complex multifactorial disorder that is usually caused by the combined influence of many genetic and environmental risk factors of small effect (Bishop 2009). In sharp contrast to Down syndrome, there is usually no clear dividing line between normality and abnormality in its aetiology, and although SLI is influenced by genes, it is not possible to diagnose it using a genetic test.

The literature on brain correlates of SLI tells a similar story. Although striking abnormalities such as developmental cortical malformations are sometimes noted (De Vasconcelos Hage *et al*. 2006), more usually, where correlates of SLI are found on structural or functional imaging, they tend to be subtle and not always consistent from study to study (Leppänen *et al*. 2004). Overall, we are not in a position to diagnose SLI from brain scans. Of course, we cannot rule out the possibility that with new techniques and better data, we might achieve what many regard as the Holy Grail: a system for diagnosis of neurodevelopmental disorders based on biomarkers rather than behaviour. However, we are a long way from achieving that goal: Even where biomarkers are found, they are seldom specific to a particular condition (Leonard *et al*. 2008).

It might be thought that such evidence invalidates any attempt to apply a ‘medical model’ to children's language problems, but as Taylor and Rutter (2008) pointed out, a view of medicine as involving only categorical syndromes with single causes is unrealistic. Medical conditions such as hypertension, obesity and kidney disease are all diagnosed on the basis of measures that are above cut-off on a quantitative scale. This may identify a group of people who are heterogeneous: hypertension can arise for a host of different reasons, and may not have any one clearly defined cause; rather it results when there is a constellation of genetic and environmental risk factors. There will often be co-occurring problems: the obese individual is likely also to suffer from other physical and psychiatric problems. Nevertheless, we find it worthwhile identifying these conditions because, when a person falls on the extreme of a normal distribution, they are at risk of further problems and may be helped by specific interventions. Those interventions may include pharmacological agents, but may also involve lifestyle recommendations such as changes in diet and exercise. The analogy with children's language impairments should be evident: in applying a label such as SLI, we are not assuming that the child has a distinct medical syndrome, that all children so labelled are the same, that language is the only problem that is present, that the child is qualitatively different from others, or that non-medical interventions will be ineffective. We are, however, acknowledging that biological, as well as environmental, factors affect a child's language development.

## 4. What are appropriate criteria for identifying children's language problems?

There is no simple answer to this question because the specific criteria that are optimal will vary with the purposes of diagnosis (Bishop 2004). In some contexts, we may give most weight to evidence of poor skill on a test of a specific component of language processing, such as grammar or verbal memory. In other situations, the key issue will be how well the child is functioning in everyday life, at home and at school. A key point is that the specific purpose of a labelling system will dictate which criteria are used. We will first consider what types of information are typically considered when evaluating a child's difficulties, and then discuss how these may be applied depending on the purpose of diagnosis.

### Information used in diagnosis

The traditional approach to identifying SLI has involved three components of diagnostic criteria, which together are intended to select children whose language difficulties have no obvious cause:

#### Evidence of significant language impairment

Although this may seem simple enough, assessing and quantifying language raises numerous questions. For instance, should we measure language using standardized tests, and if so which ones? Tomblin *et al*. (1996), for instance, made a case for excluding phonological impairment (a linguistically based speech-sound disorder) or pragmatic impairment in their diagnostic system for SLI, focusing instead on vocabulary, grammar and narrative skills. It could, however, be argued that phonology or pragmatics are part of language that should be included in a definition of SLI. Another question is what cut-offs should be used? Traditionally, scores that are at least 1 or 1.5 SD below the population mean are regarded as evidence of impairment, but this is an arbitrary criterion.

We also have the thorny problem that language tests may not capture important aspects of everyday communication. Several studies have shown that children who are judged to have language difficulties by parents or professionals are not necessarily the same children who are selected by language tests (Law *et al*. 2011, Roy and Chiat 2013, Tomblin *et al*. 1997). If we rely on parents or teachers to identify which children need help, we need to be aware that factors such as social background, as well as the type of language difficulty, may determine whether problems are detected (Bishop and McDonald 2009, Tomblin *et al*. 1997). This is potentially problematic: we do not want to waste scarce resources on children who are not experiencing any day-to-day problems, but some children with hidden language problems—especially those affecting comprehension—can get missed unless formal language testing is used. A key point here is that a language problem may not always look like a language problem: an underlying comprehension impairment can present as poor academic attainment, impaired social interaction, or behavioural difficulties (Cohen *et al*. 1998).

#### ‘Cognitive referencing’

‘Cognitive referencing’ is the practice of evaluating a child's language skills in relation to the level of nonverbal ability, rather than chronological age (Cole and Fey 1997). Implicit in this criterion is the notion that a child with a mismatch between language and nonverbal skills is different from one whose poor language is at a similar level to nonverbal ability. However, as discussed further below (Question 5), there is no good evidence that this is the case (Tomblin 2008). Accordingly, this criterion is now largely discredited, and the more usual approach is to require only that the child achieve some minimum level of nonverbal ability (though there is no consensus about which nonverbal test and which cut-off to use).

#### Exclusionary criteria

The use of exclusionary criteria seems simple enough: we wish to separate those children for whom there is a known cause of language problems, from those that are unexplained. In practice, however, this is not always easy.

##### Genetic syndromes

A child with a known genetic syndrome, such as Down syndrome, would not usually be categorized as a case of SLI, because there are usually widespread cognitive deficits extending beyond language –though language skills tend to be disproportionately worse than nonverbal ability (Laws and Bishop 2004). But what about Klinefelter syndrome (47, XXY karyotype)? Children with this chromosomal constitution often have a cognitive profile that is similar to that seen in SLI, with depressed verbal skills in the context of normal nonverbal ability (Bishop and Scerif 2011). Should they therefore be included as cases of SLI? The answer, as always, varies according to the purpose of diagnosis, as will be discussed further below.

##### Hearing loss

Another example that may be less simple than it appears is the case of the child with moderate to profound sensori-neural hearing loss. A permanent hearing loss of this level of severity will typically impair acquisition of oral language, and may lead to a pattern of language difficulties similar to that seen in normally hearing children with SLI (Bishop 1983). Nevertheless, there is still wide variation in the extent of language problems. This was demonstrated in a study of children receiving cochlear implants, some of whom had language problems that were far more severe than was usually seen with that degree of hearing loss (Hawker *et al*. 2008). The authors suggested that they might have both hearing loss and risk factors for SLI. This interpretation was supported by a subsequent study showing evidence of increased language impairment in the normally-hearing siblings of cochlear implant users with disproportionate language impairment (Ramirez-Inscoe and Moore 2011). There are also hearing-impaired children who fail to master sign language, despite adequate opportunity to learn, who can be regarded as having a SLI for sign (Mason *et al*. 2010).

##### Social deprivation

**Figure 1:**
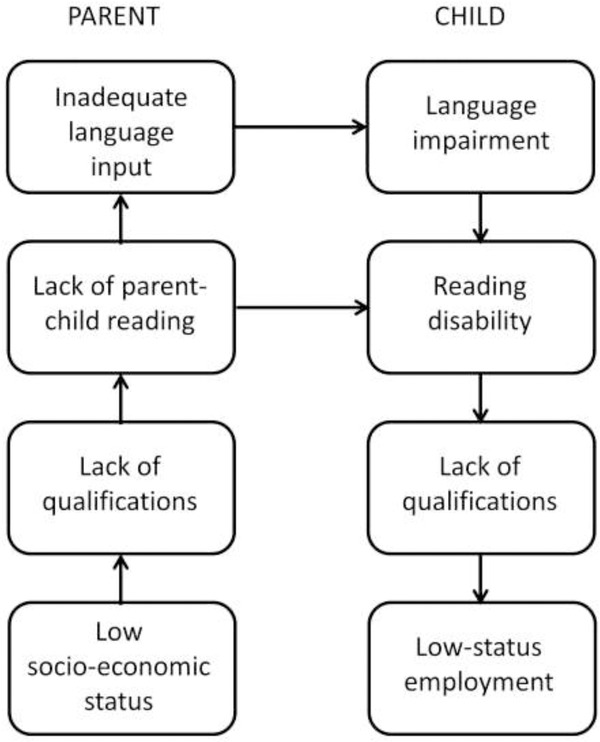
Causal model in which there is a direct link from communicative behaviour in the parents to language impairment in the child.

**Figure 2:**
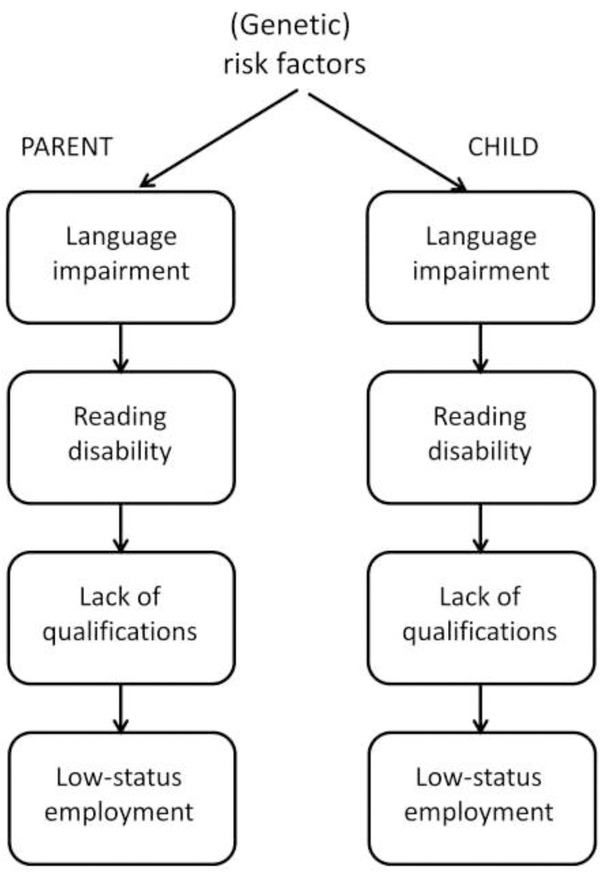
Causal model in which shared genes account for association between socioeconomic factors in the parents and language impairment in the child.

Roy and Chiat (2013: 131–132) noted that SLI can be interpreted as ‘poor language performance that cannot be explained by limitations in a child's language experience’, but just how realistic is it to identify cases where language problems are due to such limitations? My view is that, if we set aside cases of extreme neglect, it is not. While it is well-established that there is a positive association between social disadvantage and children's language skills (Letts *et al*. 2013, Schoon *et al*. 2010), it is seldom possible to disentangle the causal paths behind this association. Social deprivation effects could arise because poor language input from parents leads directly to language difficulties in their children (Leffel and Suskind 2013, Pickstone *et al*. 2009), as shown in figure 1. However, twin studies suggest a different interpretation of the association, namely that parents and children share genetic risk factors for language impairment (Bishop 2006b). Factors such as low socioeconomic status and parental educational level are not the independent environmental factors that they are often assumed to be: they can be *consequences* of language impairment. This is amply illustrated by follow-up studies of language-impaired children. We know that when they grow up, children who have language problems have poorer educational and employment outcomes than those who do not (Johnson *et al*. 2010, Whitehouse *et al*. 2009b). As adults, they are therefore likely to have a lower educational level and lower socio-economic status than other people. Consistent with this, parents of children have, on average, poorer language and literacy skills than control parents (Barry *et al*. 2007, Law *et al*. 2009). We could thus have an association emerging between lower socioeconomic status and poor educational attainments in the parents and language difficulties in their children even if there were no causal route from parental language to child language, simply because children share 50% of genetic makeup with their parents. If a parent has heritable language impairment, his or her child will also have a higher genetic risk for SLI. Figure2 shows the causal chain suggested by this account, and contrasts it with the causal route that is typically assumed to account for the association (Figure1). The shared causal factor responsible for the association is labelled here as ‘(genetic) risk factor’ because of the evidence that language impairment is often heritable, but there could also be environmental risk factors that operate in the same way.

Of course, the different causal mechanisms shown in figures 1 and 2 are not mutually exclusive, and it is likely that in many children there is a mix of biological and environmental causes involved. It would be unwise, however, to assume that a low educational level of parents is the sole and direct cause of language difficulties in children in cases where there is social disadvantage. A distinction between language problems with environmental versus biological causes would be more justifiable if we could demonstrate some differences in the nature and pattern of language problems for children from different social backgrounds, or if they responded differently to intervention. However, to date, I am unaware of any good evidence of that kind, and indeed, Roy and Chiat (2013) found that language-impaired children with high or low SES had similar language profiles.

### Different goals of diagnosis

#### Deciding who gets intervention

In clinical settings, our principal goal is to identify children who will benefit from intervention. In this context, functional disability—evidence that the child's problems are interfering with everyday life or academic attainment—is likely to be at least as important as language test scores. However, as noted above, we need to be alert to the possibility that the child's difficulties may not be obvious, especially if they principally involve comprehension.

A further point relates to the discussion of exclusionary criteria, above. In this particular context, it is not clear that strict use of exclusionary criteria is justified, unless there is good evidence that the child has difficulties that would not respond to intervention. For instance, a child with Klinefelter syndrome may benefit from the same kind of intervention as a child without any additional diagnoses.

In the final analysis, we should be identifying those children who will benefit from targeted help. Unfortunately, there is a dearth of high-quality research on effectiveness of intervention in this area, and this makes it difficult to devise well-motivated, evidence-based criteria.

#### Epidemiology and audit

Knowing how many children are affected with a condition is important for planning resources, and for identifying causal factors that may vary across time and place. Lack of an agreed set of criteria for language impairment makes comparisons of prevalence rates problematic. A widely adopted solution is to take a statistical definition, selecting children whose scores on a language test are below some specified cut-off, e.g. the bottom 10%. However, such a criterion will select a constant, and arbitrary, percentage of children, and may relate only poorly to measures of functional impairment. Tomblin *et al*. (1997) noted that prevalence rates are not entirely predictable from statistical cut-offs used for diagnosis, because some of those falling below cut-off will meet exclusionary criteria. In addition, if we use tests that are normed for a representative population, we can consider how rates of impairment vary within substrata of that population. Nevertheless, use of statistical cut-offs creates the same problems that are seen when we try to set standards for determining levels of poverty, or prevalence of short stature. Income, height or language ability of the whole population could improve substantially, but a statistical cut-off will still select a specific proportion, such as the bottom 10%. We can only avoid this by identifying an absolute anchor point for impairment. For instance, Rice (2000) argued against purely statistical criteria, maintaining that some key differences between impaired and unimpaired children are not readily assessed on tests that generate normal distributions of scores. She suggested that, in English-speaking children, a failure to use aspects of grammatical morphology reliably by 5 years of age can be used as an indicator of language impairment—a view supported by a recent study by Redmond *et al*. (2011). The field would benefit from additional specification of absolute criteria for language skills that should be mastered at given ages to allow us to escape from the circularity inherent in statistical definitions. This is a challenging task, which may require different solutions for different languages.

#### Research on correlates of language problems

If the goal is to find the underlying neurobiological or cognitive bases of language problems, then it may be more important to select a group of children who are homogeneous in terms of their language profile, rather than to focus on those with the most severe functional impairments. Furthermore, to isolate correlates of language deficits, we may want to focus on children who do not have any additional problems. Such pure cases are, however, rare, and not likely to be representative of children who are seen in clinical contexts, where co-occurring problems are the rule rather than the exception (Dyck *et al*. 2011).

#### Research on genetics

When doing genetic studies it might seem sensible to stick with published clinical criteria, such as those in ICD-10 (World Health Organization 1992) or DSM-5 (American Psychiatric Association 2013). For genetic studies it would certainly make sense to use exclusionary criteria to select out children with a known organic disorder that could lead to language problems, such as a chromosome anomaly, neurological disease or cochlear damage. But in other regards, a focus on ‘pure’ disorders has proved counterproductive. Relatives of children who meet stringent diagnostic criteria often have a ‘broad phenotype’, i.e. milder versions of the same problems which would not usually qualify for a diagnosis (Barry *et al*. 2007). In addition, they may have other disorders, such as autistic features, or low nonverbal ability (Bishop 1994). A focus on textbook cases can therefore be unhelpful in uncovering patterns of familiarity (Lewis *et al*. 2006). Instead, we may get clearer results if we can identify ‘endophenotypes’, i.e. measures that relate more closely to the underlying neurobiology of the condition (Gottesman and Gould 2003).

Another point emerging from genetic studies is that heritability of language impairment can vary depending on how it is defined. Bishop and Hayiou-Thomas (2008) found that alternative ways of identifying language disorder gave very different results in analysis of a twin sample. Genetic influence on impairment was marked only for children who attracted parental or professional concern. For children who had low scores on language tests but no clinical referral, there was little evidence of genetic influence. This suggests that overt problems with speech production and/or expressive language—which tend to be readily noticed and so lead to clinical referral—are more heritable than weak vocabulary, which does not attract concern unless accompanied by other difficulties.

## 5. Does it make sense to focus on ‘specific’ problems with language?

It is often assumed that we should distinguish children whose language difficulties can be attributed to a known cause from those who have unexpected, unexplained language problems. The notion of a ‘specific’ impairment has been operationalized by requiring a discrepancy between impaired language function and normal nonverbal ability—something which was part of diagnostic criteria for specific learning disabilities for many years. The discrepancy criterion captured the notion that the impairment was unexpected and unexplained: whereas there was an assumption that language deficits were unsurprising in a child who had more global intellectual difficulties. However, this rationale has not been supported by evidence in either language or literacy problems. While it is true that verbal and nonverbal impairments often co-occur, it is not the case that nonverbal ability sets a limit on language development (Bishop 2004, Tomblin *et al*. 1996). Indeed, it is possible to find children whose performance on language tests is much better than their performance on nonverbal tests—the opposite pattern to what is seen in SLI. Furthermore, inclusion of discrepancy criteria in diagnostic formulations can be a barrier to progress in studies of aetiology. For instance, Bishop (1994) found that twin data were more interpretable if children were categorized according to language deficits, regardless of nonverbal ability, than if a conventional diagnosis of SLI were used. In short, where low nonverbal ability accompanies poor language skills, it should be seen as a correlate rather than an explanation.

One setting where use of nonverbal IQ criteria can sometimes be justified is in research contexts where the goal is to identify specific correlates of poor language learning. For instance, poor phonological awareness is a well-established correlate of poor reading, regardless of IQ level. If, however, this had been discovered in children whose poor reading was accompanied by low nonverbal IQ, it is unlikely its significance for reading would have been appreciated. It would instead have been regarded as part of general developmental delay. In the field of oral language impairments, demonstration of problems with procedural learning (Lum *et al*. 2013), grammatical morphology (Bishop 2013, Rice 2000) or nonword repetition (Graf Estes *et al*. 2007) are far more striking when seen in language-impaired children of normal nonverbal ability, than if demonstrated in those with more general learning difficulties.

## 6. Are language problems distinct from other neurodevelopmental disorders?

In the past, research on different neurodevelopmental disorders proceeded largely independently, but there is growing awareness of considerable overlap between different conditions. First, it is evident that many children with SLI meet criteria for developmental dyslexia and vice versa (Bishop and Snowling 2004). The overlap was for many years not appreciated, because reading and oral language problems are usually dealt with by different professional groups: psychologists or educators for reading problems, and speech–language therapists for language problems. As the evidence grew for close relationships between disorders of written and spoken language, people started to ask whether SLI and dyslexia were the same condition presenting at different points in development. Bishop and Snowling (2004) concluded that the reality was more complex, with different children showing different combinations of underlying problems, which may be restricted to phonological processing in some cases, or extend to broader aspects of oral language in others. The message, however, is clear: it does not make sense to create a sharp division between oral and written language in any diagnostic system, because the two go hand in hand (Snowling and Hulme 2012).

There are also high rates of co-occurrence between language problems and a range of other neurodevelopmental disorders, notably speech sound disorder, ADHD, developmental dyscalculia, and developmental coordination disorder (DCD: more informally termed ‘developmental dyspraxia’) (Bishop and Rutter 2008). We still do not know the reason for these overlaps, but it seems likely that they occur because the same environmental or genetic factors that increase risk for language problems also increase risk for other neurodevelopmental disorders. Should we refer to language impairments as ‘specific’ when they occur together with these other conditions (Hill 2001)? It comes down to how words are used. If by ‘specific’ we mean that the child has no problems other than with language, then this is clearly an inappropriate term if ADHD or DCD is also present. If, however, we take ‘specific’ to mean ‘idiopathic’ or ‘functional’, i.e. with no known cause, then the term is still applicable, because the co-occurring condition is not an explanation for the language problems.

**Figure 3:**
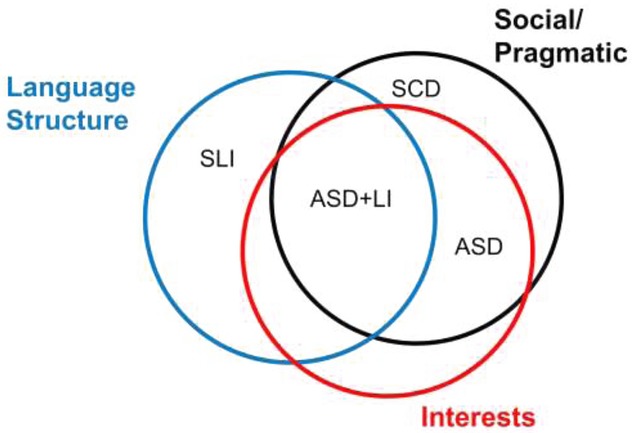
Relationship between social communication disorder (SCD), specific language impairment (SLI) and autism spectrum disorder (ASD) in DSM-5. Unlabelled regions of the Venn diagram do not correspond to specific diagnostic labels, though it is likely that some children would fall in these regions.

Autism spectrum disorder (ASD) is of particular interest, because traditional diagnostic criteria exclude a diagnosis of SLI when ASD is present, yet it is clear that a subset of children with ASD also have language difficulties that are similar to those seen in SLI (Tager-Flusberg and Caronna 2007). This has led researchers to subdivide children with ASD into those with and without additional language impairments (Lindgren *et al*. 2009). Even more complex for any diagnostic system are children who appear to occupy a position that is half-way between ASD and SLI. These are children who have problems with pragmatic aspects of communication, yet do not have the repetitive behaviours and restricted interests characteristic of autism. In some cases they also have the kinds of grammatical and phonological difficulties typical of SLI. The solution in DSM-5 has been to create a new category of social communication (pragmatic) disorder (SCD) for these children (figure 3). Norbury (2014) has pointed out a number of problems with this solution: it treats SLI and SCD as different conditions, though often there are overlapping impairments in the two groups; it bases diagnosis on aspects of social communication for which reliable and valid assessments are lacking; and there is a risk that children may end up with no suitable intervention if no professional group feels responsible for meeting their needs.

## 7. What labels have been used for unexplained language problems?

**Figure 4:**
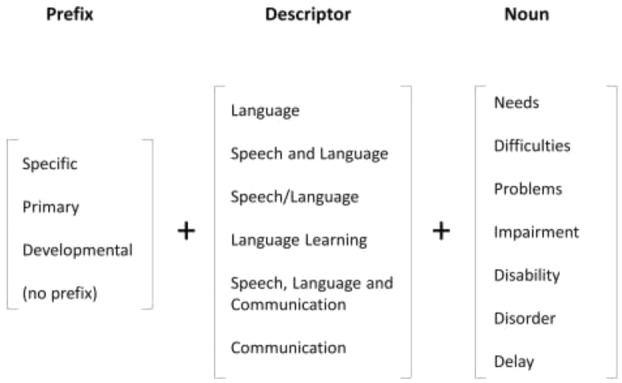
Possible terminology for children with unexplained language problems: 130 of the 168 possible combinations of a prefix, descriptor and noun were found on a literature search using Google Scholar.

The diagnostic mayhem affecting the field of children's speech and language impairments is illustrated in figure 4. Most labels consist of some permutation of the terms shown in the figure, i.e. an optional prefix (specific, primary, or developmental), a reference to the language domain, and a noun that indicates we are identifying a child with a problem. A search on Google Scholar for each phrase for the period 1994–2013 revealed that 130 of 168 possible combinations had at least one return. Two additional terms that were counted were ‘developmental aphasia’ and ‘developmental dysphasia’. Terms with more than 600 returns are shown in table2. This reveals a massive problem: not only are there numerous possible terms, but also they can have different meanings. By far the commonest terms were ones with no prefix, but their use was not restricted to children with unexplained language problems. Indeed, the terms, ‘communication delay’ and ‘communication problems’ were widely used to refer to electronic systems. ‘Communication disorder’ identified papers on language or communication difficulties of adults with Parkinson's disease or acquired aphasia, and children with Down syndrome. ‘Language needs’ often referred to second-language learners. It is of concern that ‘language disorder’ is the term used in DSM-5 to refer to children with unexplained language problems, yet is effectively useless in a literature search because it is far too general.

**Table 2:** Number of returns for terms with at least 600 returns on Google Scholar, search date range 1994–2013

Label	Number of hits
*Communication problems^a^	56 739
*Communication needs	40 632
*Language problems	40 427
*Language difficulties	32 610
*Communication difficulties	32 530
*Language needs	21 139
Specific language impairment	18 850
*Communication delay^a^	17 594
*Language impairment	16 663
*Language disorder	16 208
*Language delay	14 786
*Communication disorder	7061
*Communication impairment	4611
*Language disability	3738
Developmental language disorder	3509
*Speech and language difficulties	2602
*Speech and language disorder	2584
*Speech and language problems	2486
*Communication disability	2376
Developmental aphasia	2097
*Speech and language impairment	2081
*Speech and language delay	1781
Developmental dysphasia	1772
*Language learning needs	1758
*Speech/language impairment	1718
*Language learning difficulties	1595
*Language learning problems	1328
Developmental language delay	1310
Developmental language impairment	1105
*Language learning disability	783
*Speech/language disorder	685
*Speech, language and communication needs	673
*Speech/language problems	646

Notes:

* Counts for these terms after subtracting cases preceded by ‘specific’, ‘primary’ or ‘developmental’, which are counted separately.

a Frequently used to refer to electronics systems.

If we focus just on terms that have a prefix that distinguishes childhood language problems of unknown origin, then table2 shows that the term ‘specific language impairment’ is the most commonly used: five times more common than the next in the list, ‘developmental language disorder’. As noted, however, there are objections to the label SLI, centring around the word ‘specific’. This implies that the language problems occur in the context of otherwise typical development and, this is only rarely the case.

Further confusion surrounds the use of terms such as ‘speech and language’ or ‘speech/language’, because they are ambiguous. They could be used to group together children with speech *or* language difficulties, or to refer to those who had problems in both domains. Indeed, ‘speech’ is a term used with various meanings, and can include those who have articulatory difficulties for structural or neurological reasons (e.g., cleft palate or cerebral palsy), or for cases of ‘speech sound disorder’ which are not attributable to sensori-motor causes, and may be better characterized as language problems affecting the phonological domain. ‘Communication’ is another alternative which seems too broad to be useful: although sometimes used with more specific meanings, it potentially includes nonverbal communication and social interaction, as well as language and speech.

Another part of terminology that can be controversial is the third column in figure 4: how problems are referred to. Should we talk about language impairment, disorder, disability, difficulties, needs or delay? In practice, these are often treated as synonyms, yet they have different connotations and political implications. The term ‘disability’ was introduced as part of ‘specific learning disability’ in the United States in the 1960s to refer to children who had difficulties learning despite being of normal intellectual capacity. As Waber (2010) noted, there were legal ramifications in the choice of terminology. ‘Learning disability’ drew parallels with other disability conditions, and led to provisions being made in law for federal funding for education and research for affected children. ‘Disorder’ is widely used in medical contexts to refer to neurodevelopmental problems of no known cause, including autistic spectrum disorder and developmental coordination disorder. ‘Language disorder’ is the term used in DSM-5. However, both ‘disability’ and ‘disorder’ are disliked by some practitioners because they are seen as emphasizing abnormality rather than quantitative differences between children, and they focus attention on problems within the child. The acronym LD is also ambiguous, being used for learning disability (which means intellectual disability in the UK but *specific* learning disability in other English-speaking countries).

The term ‘delay’ is fairly common but highly ambiguous. A parent who is told that their child's development is delayed might reasonably assume that it will follow a normal course but at a later age than usual. ‘Language delay’ is indeed sometimes used this way, to refer to late-talking toddlers who subsequently catch up with their peer group. However, another use is to draw an implicit contrast with ‘language disorder’, but agreed criteria for making this distinction do not exist. One view is that a child with language delay will have language that in all respects resembles that of a younger, typically developing child, whereas a child with language disorder will have an abnormal profile. Yet in practice, children who have selective problems with specific components of language (potentially cases of ‘disorder’) have a better prognosis than those with a more even depression of language skills (Bishop and Edmundson 1987), which seems counterintuitive. And in addition, it is clear that, at least in the research literature, ‘delay’ is seldom used with such a distinctive meaning: more often, it is just another synonym for below-age-level language skills.

The term ‘impairment’ has a clear definition in the World Health Organization's (1980) classification of impairments, disabilities and handicaps, but in the context of children's language problems it is used with a rather different meaning. It does not refer to physical impairment, but rather to poor performance on a measure of language skill. Bishop (2004) suggested that, in contrast to ‘disability’, ‘impairment’ can be used without any implication that there is an impact on functioning in everyday life. For instance, some children who do poorly on a test of nonword repetition do not have evident problems in everyday communication or academic achievement. Nevertheless, an impairment in nonword repetition can run in families, and may put the child at risk for language or literacy problems if it occurs in combination with other risk factors (Bishop 2006a, Snowling 2008).

In the UK, ‘needs’ began to be used in educational contexts after the Warnock Report (Warnock 1978), which introduced the term ‘special educational needs’ (SEN) to break away from dichotomizing children into the ‘handicapped’ and everyone else. The report noted that up to one in five children were likely to require some form of special educational provision at some point, and children with language difficulties were explicitly included in this group. The term ‘needs’ represented a move away from a focus on deficit—what the child or young person could not do—to what was required to provide learning opportunities and support academic progress. It seems, though, too weak a term to convey the major, long-term language deficits that affect some children. Similar criticisms may be made of the terms ‘problems’ and ‘difficulties’: everyone has ‘needs’ and encounters ‘problems’ and ‘difficulties’ in life, but other people may feel little obligation to do anything about this if they are just regarded as normal challenges of everyday existence.

## 8. What are the consequences of the lack of agreed terminology?

In many respects, diagnostic dilemmas in the field of children's language problems are similar to those for other conditions such as reading or attentional difficulties: In all cases, there are questions about the appropriateness of a medical model, difficulties in specifying cut-offs to define disorder, and overlaps between different conditions. However, there is one problem that is particular to the domain of language, and that concerns the lack of an agreed label. In this regard, SLI is very different from developmental dyslexia. Just as with SLI, children with a diagnosis of developmental dyslexia are quite variable in both the severity and the profile of their literacy problems, there is no clear dividing line between dyslexia and normal variation, the aetiology is complex and multifactorial, and there is no good biomarker of the condition. Accordingly, the label ‘developmental dyslexia’ has been repeatedly attacked over the years by those who have pointed out how misleading it is in implying that we are dealing with a homogeneous syndrome with a neurological basis. This case has been made again with renewed vigour in a recent review of evidence by Elliott and Grigorenko (2014). They argue that ‘developmental dyslexia’ has no validity, and they make the case that persistent use of the term does a disservice to other poor readers who are denied the extra resources and legal protection that are afforded to those with this label. Nevertheless, the term is likely to weather this attack, just as it has withstood previous assaults (Rutter and Yule 1975, Stanovich 1994). The evidence comes again from bibliometrics, where one can trace changing terminology used at different points in history. Attempts to introduce alternative terms such as ‘specific reading retardation’ (Rutter and Yule 1975), ‘reading disorder’ (American Psychiatric Association 1994) or ‘language-based learning disabilities’ (American Speech–Language–Hearing Association n.d.) have been ignored by the majority of people: In the bibliometric database used by Bishop, the term ‘dyslexia’ accounted for 93% of research papers on children's reading problems in 1985–89, rising to 99% from 2000 onwards. Quite simply, in spite of its poor validity, the term is a successful meme (Kamhi 2004). One reason for this success may be that ‘dyslexia’ emphasizes the positive consequences listed in the second column of table1, with some children and young people talking of a sense of relief at receiving the diagnosis (Ingesson 2007) and some claiming that dyslexia has positive attributes—but see Seidenberg (2013).

There is nothing comparable for children with unexplained language problems. If they are provided with a label, it will probably be one that most people have not heard of, and it is unlikely to have any positive connotations. The lack of agreement about terminology means that many will either misunderstand the condition or doubt its reality.

The terminological confusion also has a detrimental effect on research (Bishop 2010). It is very difficult to assemble information from the research literature because one must search using multiple different terms, some of which will capture a large amount of irrelevant material. Any attempt to apply for research funding is hampered by the need to first explain to funders what the condition is that one is researching: it cannot be assumed that they will have any notion of the nature, prevalence, personal implications or social impact of children's language difficulties. The amount of research funding, and the number of published papers on unexplained language problems is considerably less than one would predict from knowledge of the frequency and impact of such problems (Bishop 2010): It seems likely that lack of agreed terminology plays a significant role in this deficit.

## 9. How might we enhance positive consequences, and avoid negative consequences, of labelling?

I have argued in favour of an agreed label to refer to children with unexplained language problems, but noted too that there can be unintended negative consequences of using labels. How can these be averted? First, a child who receives such a label should automatically qualify for an evaluation by a language specialist—usually a speech and language therapist—who would aim to identify barriers to language learning and put intervention in place to counteract or compensate for these. Note the mention of compensation: there are rather few kinds of language intervention that have been validated as effective in clinical trials for improving serious language deficits, especially those involving comprehension (Law *et al*. 2004). This does not mean that we should stop trying to develop interventions, but it does imply that one role of the therapist will be to work with children and their teachers to develop effective strategies for coping with problems and accommodating to them. The second recommendation is more radical: it is that any child identified with unexplained language problems should also undergo an evaluation to identify areas of strength: activities they enjoy and have the possibility of succeeding at. These could, for instance, involve sports, art, cookery, graphic design, horticulture, working with animals or music. Realistically, we would not expect all children to have hidden talents, but we should move from a frame of mind that is solely focused on deficits, and attempting to ‘fix’ these so the child can gain academic credentials. We have ample evidence that most children with language learning impairments (LLIs) have difficulties that persist into adolescence (Conti-Ramsden and Durkin 2008, Stothard *et al*. 1998) and beyond (Clegg *et al*. 2005, Johnson *et al*. 2010, Whitehouse *et al*. 2009a). We should therefore be thinking more about how to enable children to be successful citizens, and this may require us to move away from narrowly conceived academic ideas of success.

## 10. What terminology should we adopt?

I have argued that we need an agreed terminology to describe children whose language is well behind age level for no obvious reason. As Tomblin (2008: 95) put it: ‘language disorder represents a situation in which the child is unlikely to be able to meet the socially defined functional expectations either currently or in the future because of his or her current or future language abilities’. We know that when language problems persist into school age, the outcomes for children are usually poor. While they may benefit from school-based programmes designed to foster language development in all children (Law *et al*. 2013), this is unlikely to be sufficient to overcome the academic and social difficulties that ensue when language expression and/or comprehension are well behind that of the peer group.

Labels can have negative consequences, but the consequences of avoiding labels can be worse. Without agreed criteria for identifying children in need of additional help, and without agreed labels for talking about them, we cannot improve our understanding of why some children fail, or evaluate the efficacy of attempts to help them. The fact that language difficulties do not constitute a specific syndrome is not a sufficient reason to abandon labels.

The current situation, with myriad different definitions and labels, is unsustainable. Having an unconstrained set of descriptive terms is just as bad as having no labels at all. It hinders communication, prevents cumulative research, and introduces ambiguity into decisions about who merits intervention—ambiguity that can easily be exploited when it is politically expedient to do so.

Although I have argued that the purpose of diagnosis will determine the ideal diagnostic system, there needs to be contact between different approaches: those working in education, in speech–language therapy and in research need to have a common vocabulary that allows information to be exchanged between these disciplines.

One point that is often overlooked when devising classification systems is the importance of having a label that is a good term for use with internet search engines. In this regard, general terms, such as ‘language disorder’ are too nonspecific to be useful; although they can be applied to unexplained language problems, they are also used descriptively for adults as well as children with a wide range of aetiologies. The term ‘speech, language and communication needs’ (SLCN), which is widely used in the UK in educational contexts, is also too general, as it includes both speech and language difficulties, and fails to distinguish unexplained language problems from those that can be attributed to a known cause. While there may be situations when it is not necessary to distinguish problems by type or by aetiology, very often this distinction is of practical importance in education, as well as being crucial for research.

Of the less general terms in current use, SLI is by far the most common in academic settings, though it is less widely used in clinical and educational practice in the UK. A case could be made for retaining this term, to maintain continuity with the past. It has, however, one drawback, which is that the ‘specific’ part of the label has been criticized for being too exclusive. If we take ‘specific’ to mean that the child (1) has a substantial discrepancy between language and nonverbal ability and (2) has no other neurodevelopmental difficulties, then a vanishingly small proportion of language-impaired children would be included as cases of SLI. In practice, the criteria have loosened over the years, and it is no longer common to interpret SLI as requiring a large mismatch between verbal and nonverbal skills: rather children are included if they have notable language difficulties in the context of broadly normal-range nonverbal ability—usually interpreted as having a nonverbal IQ of at least 80 (though some use other cut-offs, ranging from 70 to 85) (Tomblin *et al*. 1996). Furthermore, the presence of other conditions such as dyslexia, ADHD, or DCD would not usually be regarded as precluding the diagnosis of SLI. So we could just agree to keep the term SLI, but to adopt laxer criteria that did not specify an absence of other neurodevelopmental problems, and that require only that nonverbal IQ should be broadly within normal limits. This corresponds to usage by the American Speech–Language–Hearing Association (2008). In addition, we might want to restrict the use of SLI to children who have a functional impairment affecting everyday communication, social interaction, behaviour, and/or academic attainment.

We also need to reach agreement about a common set of language components that should be included in a language assessment for SLI. In clinical practice, the choice of measures can be quite arbitrary, but is of potential importance: it could, for instance, determine whether children meeting DSM-5 criteria for social communication disorder are included or not. One approach would be to include those aspects of language that reliably have emerged as good ‘markers’ of SLI (Bishop 2004, Conti-Ramsden 2003, Redmond *et al*. 2011). These mainly involve aspects of language structure and verbal memory, rather than language content or use.

SLI is not, however, the only terminological option open to us. An alternative term that would be precise enough to be useful, without having unwanted connotations of specificity is primary language impairment (PLI). This term is not in widespread circulation—it had only 362 returns on my Google Scholar search—but it has been used in two contexts: first, when identifying language impairments that are not accounted for by bilingualism (Kohnert 2010) and second as a more inclusive term to refer to language difficulties that are not secondary to another condition, without requiring a discrepancy with nonverbal ability (Boyle *et al*. 2007). One drawback is that the acronym PLI has potential for confusion with ‘pragmatic language impairment’ (Bishop 2000), though it could be argued that this is not important, given that ‘pragmatic language impairment’ was never part of any official diagnostic framework, and DSM-5 has now coined ‘social communication disorder’ which covers the same territory.

Another option would be to revert to a term such as ‘developmental language disorder’, which was more commonly used some 20–30 years ago. As noted above, ‘disorder’ is disliked by some because it has medical overtones and implies qualitative rather than quantitative differences between children. ‘Developmental language impairment’ would be another possibility, which is already in circulation (table2).

Finally, another option would be the term ‘language learning impairment’ (LLI). Like PLI or developmental language disorder, this avoids confusion with more general language problems from known aetiologies, without implying that the language problems occur in isolation. It also emphasizes that this is a kind of learning difficulty, rather than reflecting a lack of progress due to inadequate stimulation. This is the term that we settled upon when considering how to refer to unexpected language difficulties in an internet campaign to raise awareness: Raising Awareness of Language Learning Impairments (RALLI) (Bishop *et al*. 2012). However, only time will tell whether it becomes more widely accepted, or joins the long list of possible labels that serve only to add to confusion in this field. Changing a label should not be undertaken lightly, as it can break links with previous knowledge: this is why in the RALLI campaign we still use ‘specific language impairment’ in many of our videos, as this is a better-known label, and more likely to be used as a search term. Only by having discussions with a wide range of stakeholders can we hope to reach a consensus on terminology.

Many of the points made by Frances (2013) in his DSM-5 critique would apply equally to our deliberations about a label such as SLI. We should heed his warnings about unintended consequences of diagnostic inflation and medicalization of normality. But we should note too his comments about the importance of diagnostic labels for those whose problems are severe, clear-cut, and unlikely to go away on their own. We must accept that we will never have an ideal nomenclature, suitable for all purposes: As Frances noted, diagnosis has a necessary place in every evaluation, but never tells the whole story. We must not reify our labels, but recognize they are a collection of ‘temporarily useful diagnostic constructs, not a catalogue of “real” diseases’ (Frances 2013: 73).

**How to cite Commentary articles**

Please use the following style:

Baird G., 2014, Lumping, splitting, drawing lines, statistical cutoffs and impairment. Commentary on Bishop, D.V.M., 2014, Ten questions about terminology for children with unexplained language problems. *International Journal of Language and Communication Disorders*, **49**, 381–415. doi: 10.1111/1460-6984.12101
