# Supplementary material for: Ten questions about terminology for children with unexplained language problems
Source: Int J Lang Commun Disord. 2014 Aug 20;49(4):381–415. doi: 10.1111/1460-6984.12101 (PMC4314704; doi:10.1111/1460-6984.12101)
Supplement: Related texts

# Other: Lumping, splitting, drawing lines, statistical cut-offs and impairment: a commentary on ‘Ten questions about terminology for children with unexplained language problems’

**Published:** 2014-08-20

**Authors:** Gillian Baird

**Affiliations:** Professor of Paediatric Neurodisability and Consultant Paediatrician, Guy's & St Thomas NHS Foundation Trust and King's Health PartnersLondon, UK

## Abstract

There is an agreed need for some classification system of language problems, but the varying views about inclusion/exclusion criteria, the lack of biomarkers and the similar problems affecting all neurodevelopmental disorders are explored.

## Main text

There have long been debates about whether some children learn language differently or are simply at the lower end of a normal continuum of language development (Leonard 1991). Much research has suggested that there is a group of children who have a real problem in learning language and that this is lifelong, that the effects are initially on understanding and speaking language, and subsequently on reading and understanding written language. Persistent deficits have been shown in aspects of language processing, such as non-word repetition and reading non-words, leading to suggestions that such skills are markers for language learning and reading problems.

The hypothesis of a developmental learning disorder affecting language is recognized in the current classification systems (ICD and DSM) alongside similar learning difficulties in motor coordination, attentional development and academic skills. For each of these the same debate has occurred over whether these disorders represent the extreme end of a normal continuum or a specific learning impairment which is different and can be separated by specific tests. Therein lies the difficulty—an absence of specific tests that clearly differentiate a disorder from a normal continuum. In all these developmental areas it becomes a matter of clinical judgement about where ‘lines’ are drawn. However, this problem is not confined to neurodevelopmental disorders and is much the same in measures of physiology such as high blood pressure, a topic in which debate over what is normal and what is a case needing intervention is equally vigorous. As Bishop points out, many of the disorders are multifactorial in aetiology. The problem with classification systems is that they impose categories upon dimensions and in the absence of biomarkers are based on surface features of behaviour, which include measures that are standardized, norm-based, etc. Bishop draws attention to the limitations of a statistical approach to defining language problems (favoured by Reilly *et al.* 2014) and also the likely inconsistency of using impairment (or concern by teacher or parent) alone as a defining feature. What is lacking are agreed inclusion criteria.

Professor Bishop has been at the forefront of research into language disorders, the reasons for them and the links with literacy, and thereby has contributed to our understanding of both the immediate and long-term problems that are experienced by children and young people with language-learning difficulties. As always, she writes lucidly. The opening vignette summarizes the dilemma for the individual child and also the perspectives of those from various services inevitably involved, whether it be speech and language, other members of the health service, the education service, and parents struggling to understand what the problem is with their child.

The fourth section, in which Bishop outlines the criteria for identifying children with language problems, is particularly helpful, highlighting the difficulties of differentiating from typical development, the problems of using cognitive ability as separating those with language impairments from those with additional learning problems; whether or not genetic syndromes should be excluded and, very importantly, the influence of language environment and/or social deprivation on language development. Bishop makes a very good case for her own view that genetic risk factors play a far larger part than is generally understood by those writing about social deprivation.

I am glad that Bishop goes on to defend the use of diagnostic classification and hence ‘labels’. The diagnostic approach has been criticized as over-medicalizing normal variation or for placing a problem within the person rather than society.

However, at its best it prompts a problem-based approach that leads the parent and then the professional to seek an answer to the question: ‘Why has my child got this problem? Is there a treatable cause?’. A classification system should have clinical, public health and research utility. It provides a language for communication with the individual and parents/carers/families and with others (health, education etc.) who will be responding to that individual's problems. It provides a framework for research and access to specific evidence-based treatments. As Bishop emphasizes in her second section, identifying specific deficits, evaluating interventions and giving parents a prognosis are all helpful correlates of taking a diagnostic classification based approach.

One danger, of course, of classification systems is that labels are reified and both clinicians and researchers stop thinking about whether this is the best way of classifying a problem. Researchers need continually to challenge the existing categories and definitions. An obvious example is the change from DSM-IV to DSM-5 in autism spectrum disorders where subgrouping into atypical autism, Asperger's syndrome etc. was found not to be consistently applied. There were no really clear distinctions between the subgroups that met critical evaluation, there were no genetic factors distinguishing subgroups. They have now been abandoned in DSM-5 and included under the more general title of autism spectrum disorders, a lumping rather than a splitting approach. This does not mean that a splitting approach may not become appropriate again, but on a different basis rather than the current DSM-IV and ICD-10 one. Reilly *et al.* (2014), I think, seek to do the same with language impairment.

Bishop's views about the ‘label’ used for those with language-learning problems are particularly pertinent to current discussions, especially her strictures about the DSM-5 label of language disorder and the need for a ‘searchable’ term. There are many problems with the term ‘specific’ which she (and Reilly *et al.*) outline, particularly given the increasing recognition of coexistence with other developmental disorders and the fact that using a non-verbal reference point is increasingly regarded as not helpful. ‘Disorder’ is the term used throughout DSM and ICD for conditions without obvious aetiology and is intended to separate any condition from its functional effect as described separately in the International Classification of Function, Disability and Health.

In conclusion, the elusive goal in language-learning problems, as in other developmental conditions, remains finding underlying neurobiological factors with the aim of selecting those who need particular interventions and compensatory strategies, rather than measuring surface features and arguing about statistical cut-offs. The biomarkers proposed have not been clearly tested in population samples. Language learning is heterogeneous, so one marker such as non-word repetition may be too simplistic and has, in any case, been more strongly linked to reading decoding with language impairment rather than being universally impaired in those with language-learning impairments. Agreed inclusion criteria for developmental language disorder recognizing heterogeneity and potential subgroups, rather than exclusion criteria, would help the clinical and research community. In the absence of biomarkers and underlying process impairment markers, the child that a parent or a teacher is concerned about is, as Bishop points out, as important to pay attention to as the child who fails test scores.

# Other: To label or not to label: is this the question?

**Published:** 2014-08-20

**Authors:** Fraser Lauchlan, Christopher Boyle

**Affiliations:** * University of StrathclydeGlasgow, UK; † University of New EnglandArmidale, NSW, Australia

## Abstract

We consider whether the use of labels is an inevitable consequence of the world of limited and finite resources in which we live and work, or whether there could be other positive reasons for using labels. We argue that it may be more worthwhile expending time and energy on intervention for children with language difficulties rather than on the diagnosis itself.

## Main text

Seven years ago, while working as educational psychologists (EPs) for a local education authority in Scotland, we wrote a paper on the advantages and disadvantages of labelling (Lauchlan and Boyle 2007). We did not expect the level of interest that would result, not only amongst the academic and professional community, but also in the media (*Times Educational Supplement* (TES) 2007). Seven years on, the labelling debate is still rife and we have been invited to comment on Dorothy Bishop's paper which outlines the arguments for the use of different terminology (or labels) for children who have unexplained language problems.

We tend to agree with Bishop's comments that the removal of labels could unfortunately lead to the removal of support for many children, and this is a consequence that no caring professional could tolerate. We certainly do not condone the stance that some may make, as a professional, not to label a child solely to make a sociological point if the consequence was that there could be no access to services for the person and family that required it. A question then: Do we accept (perhaps reluctantly) that we use labels only because the educational/health system in which we work demands it, or do we use them for other reasons? This is at the heart of Bishop's paper in our view, i.e. that there *are* other positive reasons for using labels. However, there are counter-arguments that could be made to some of the points made. Let us consider some of these.

Bishop raises the need to have clear and objective criteria that should be used to determine which children might benefit from support. Again, we agree with Bishop's statement. However, the difficulty is that there do not currently exist any clear and objective criteria for the myriad of labels that exist in education, whether it be SLI, emotional and behavioural difficulties (EBD), autism, attention deficit hyperactivity disorder (ADHD) and dyslexia, to name but a few. And that, in itself, causes major problems for parents, teachers and other professionals working in schools, such as EPs and SLTs, and it raises the question as to whether we should be using labels at all if there is not clear agreement about how they should be applied. For example, the discussion in Bishop's paper about ‘cognitive referencing’ (a mismatch between language and non-verbal skills) would appear to be an area that has the potential for various misunderstandings and misdiagnoses.

According to Bishop, the ‘discrepancy model’ appears to have been discredited now, and instead it seems that, for a diagnosis to be made, a child only has to achieve some minimum level of non-verbal ability, although the exact level that is required and which tests should be used are, as yet, unspecified. It reminds us of a similar debate that took place in the 1990s in educational psychology (and still now, amongst some) regarding the diagnosis of dyslexia. It is generally considered to have been an unhelpful and damaging debate and it could be argued (Elliott and Gibbs 2008) that if the same amount of time and resources were put into how to intervene with children with reading difficulties rather than on how to make a diagnosis of dyslexia (including whether there should be a ‘discrepancy’ between literacy and other areas of the curriculum), then we might have considerably fewer children with reading and writing difficulties across the UK. Could the same case be made about the diagnosis of children with unexplained language problems?

Taking this point further, Bishop outlines some arguments for separating those children for whom there is a known cause for the language problems from those that are unexplained (see the discussion on exclusionary criteria). We ask the question: Does it matter? Maybe it does, maybe it does not. We are unsure. However, we are more sure that by expending our limited resources on exploring the different possible reasons for children's language problems, we are inevitably taking away resources from what can be spent on the intervention, i.e. what to do about it. Bishop writes that the distinction of problems by type or by aetiology is ‘very often […] of practical importance in education’. We disagree. We would welcome some practical examples as to when this has been the case, as it is our view that, while it may be important in some cases, it is not true ‘very often’.

The problems are there and must be tackled, regardless of the cause. In some respects we argue for the theoretical approach put forward by advocates of the solution-focused method (Rees 2008), namely that more time should be placed looking to resolve the problem rather than spending too much time exploring the problem itself.

Bishop discusses research on the feelings of relief that some children have experienced upon receiving a diagnostic label, especially dyslexia, which is undeniable, but did this relief lead to improved opportunities for the child? Did his/her literacy skills improve? Did the child work harder with additional vigour upon receiving the diagnosis, or did it lead to feelings of helplessness and inevitability about their difficulties that made the child try less and less? In other words, research needs to be carried out looking at these questions, in our view, not whether the diagnostic label brought some relief to the child concerned and/or their parents.

Bishop writes that when applying labels it is not assumed that all children so labelled are the same. We would hope not, however the reality is that this is indeed what happens. While there may be numerous professionals (and academics) who do not believe that children who have the same label are the same, or should be treated the same, there are countless others who do, and this is a problem with the continued blanket use of labels, and one that needs to be tackled (see Lauchlan and Boyle 2007, for further discussion).

The overarching question that we feel should be asked when considering the use of labels is the following: ‘Will the label change the child's life for the better?’. If the answer to this question is an emphatic ‘yes’, then there is little argument that can be made against the use of the label. However, if the answer, as can quite often be the case, is ‘well, perhaps, but I'm not sure, actually maybe for this particular child, no’, then we must be extremely careful to continue to attach labels in our daily working lives in our respective professions. Let us hope that labels, if deemed necessary, are applied appropriately and always to the benefit of any recipient.

# Other: Can any label work for both intervention and research purposes?

**Published:** 2014-08-20

**Authors:** Jude Bellair, Sara Clark, Stephanie Lynham

**Affiliations:** Central London Community Healthcare NHS TrustLondon, UK

## Abstract

Discussion of the issues surrounding the current specific language impairment (SLI) label identified another pertinent question: Can any label be useful for both research and intervention purposes? In exploring the relationship between a label's purpose, parameters and terminology, we conclude that no single label is suited to both purposes, but having a clear, workable label for research purposes is vital.

## Main text

The ideas in Dorothy Bishop's article prompted much debate amongst colleagues, the questions raised being both pertinent and central to everyday practice. However, discussions continually reverted to one question not raised: Can any label be useful for both research and intervention purposes? While reflecting our response to this article, our commentary also explores this additional question.

We are speech and language therapists working in Central London who have a component of time allocated to working in specialist speech and language provisions, and who both diagnose and provide intervention to children identified as having SLI. The opinions and ideas stated are our individual views and conclusions, arrived at following discussions with colleagues.

Some questions the article raised were less controversial than others, resulting in unanimous agreement. Yes, we should be concerned about children's language problems; a group of children have unresolved language difficulties that impact on their educational attainment and social relationships. No, we should not abandon diagnostic labels; the positive outcomes of using labels for intervention and research outweigh the negatives which can be mitigated by our practice. Yes, the medical model is appropriate for unexplained language problems in children; accepting that environmental factors impact on language development, children identified as having SLI are those where purely environmental causes are excluded, meaning language difficulties have a significant ‘within child’ (biological) component.

The issues around criteria for inclusion in the currently called SLI group were more contentious. It became clear that the purpose of a label influences not only the criteria applied, but also the choice of the label itself. This lead to our central question: Is there a label that works for both research and intervention purposes?

Regarding criteria, for both purposes we agreed the requirement for evidence of significant language impairment, including elements of disordered language development or such extreme delay as to be disordered. Ideally, evidence of difficulty with language *learning* would be observed or dynamically assessed. Working predominately with children exposed to more than one language, we cannot rely on standardized scores, so when making a diagnosis performance on formal testing is interpreted carefully and considered alongside the impact on interactions and access to the curriculum. Having rigid cut-off scores as inclusion or exclusion criteria is not functional for our client population.

However, some criteria important for placement on an intervention pathway were not thought important for research purposes, and vice versa. While for research purposes, excluding or including based on the *cause* of language-learning disorders is important to eliminate variables, this is not necessarily important for allocation to a care pathway. Conversely, whether a child's language-learning difficulties were the *primary* area of difficulty for the child would be important for allocation to care pathways, but not useful for research purposes.

In our view, the presence of additional difficulties such as hearing impairment, learning difficulties or ASD should not bar children from an SLI label, assuming these additional difficulties are excluded as causing the language-learning impairment (while acknowledging research may initially need to be conducted using a restricted group of SLI children). However, for a child with learning difficulty and SLI, identifying the learning impairment as the primary need would affect the choice of intervention offered, and conversely, children who had known causes for their language-learning difficulties, such as Landau-Kleffner or extreme environmental deprivation while not having SLI, may benefit from access to the same care pathway.

When thinking about interventions and care pathways, the descriptive term ‘primary language impairment’ is useful, as it incorporates children who have language-learning impairments regardless of cause, as long as language learning is their primary need, which reflects current practice. However, as a label, it has limited use for research purposes as it encompasses too broad a group for results to be valid or useful, and is reliant on somewhat subjective decisions based on function. While intervention pathways may be similar for children with language-learning difficulties whether they are idiopathic or not, research cannot be carried out on such a disparate group, and ongoing research in this area is essential.

In conclusion, we believe that keeping the term ‘specific language impairment’ is the best option, where ‘specific’ refers to ‘idiopathic’, ‘language’ encompasses the idea of ‘language learning’, and ‘impairment’ allows for variability in functional impact. By using ‘specific’ in this way, children with co-occurring difficulties or disorders can still be identified as having SLI. As a label it would be more useful for research purposes than intervention purposes, however having a label that can work for research purposes is of paramount importance. Maintaining the link with previous research into SLI is also essential, and although current research is conducted on clinical populations that are narrower than those we see in practice (i.e. only IQ above 85, or not EAL, etc.), this would be best rectified with more research and repeating successful trials on a broader group of SLI children or those with non-idiopathic language-learning difficulties. Additionally, changing the label carries a risk of children not being referred for assessment and access to specialist services, and the consequent risk to losing current funding particularly at a time of cost savings being required.

The potential negative outcomes of retaining the SLI label are within our control to manage. Instead of using diagnostic labels for care pathways, thereby excluding children without the label from a particular level or type of support, descriptions of a phenotype such as ‘primary language impairment’ could be used instead. A label, such as SLI, can be applied to an individual's impairment but does not necessarily dictate the support and interventions required: children with SLI need differing types and levels of support. Phenotype descriptions may be more useful when writing care pathways as they group children by presenting needs rather than cause or purely a label.

In recommending retention of the label SLI, we acknowledge the work needed to build consensus within our profession as to exactly what this term means, and also in increasing awareness within other professional groups and the wider community. As a profession we need to become more precise and deliberate in our use of labels versus descriptions of difficulties. The range of terms in use on electronic media is problematic, and as professionals we need to improve our awareness and ability to guide others in their search for information. However, we feel that as a profession we are up to these challenges.

# Other: Advocating for SLI

**Published:** 2014-08-20

**Authors:** Fraser Lauchlan

**Affiliations:** University of Kansas, LawrenceKS, USA

## Abstract

The label of specific language impairment (SLI) has inspired substantive advances in our knowledge of previously overlooked but clinically (and theoretically) significant language impairments. Advocacy is needed to sustain the scientific gains, do a better job of identifying and providing services for persons with SLI, and build better screening and assessment tools suited for diverse clinical and research purposes.

## Main text

My charge is to provide commentary on the paper by Bishop. I am in general accord with her perspectives and wish to use this opportunity to expand an advocacy for the label of specific language impairment (SLI). I have three main points:

- SLI, a term coined only about 30 years ago (Leonard 1981), has inspired substantive advances in our knowledge of previously overlooked but clinically (and theoretically) significant language impairments.
- As a clinical label SLI has yet to receive widespread adoption in clinical practice, in spite of the great need for it. From toddlers to young adults, the condition is likely to go undetected, untreated and be poorly understood by the general public, teachers and physicians (cf. Bishop's overview).
- Removal of barriers to services for children and adults with SLI requires informed advocacy and the development of accurate and time-efficient methods of identification, as well as population-based studies and continued high-level experimental and longitudinal studies.

The SLI diagnostic category helped change views of typical and atypical language acquisition. Consider that in 1984 Steven Pinker wrote: ‘In general, language acquisition is a stubbornly robust process; from what we can tell there is virtually no way to prevent it from happening short of raising a child in a barrel’ (Pinker 1984: 29). In contrast, now the National Institute of Deafness and Communicative Disorders (NIDCD) includes research about SLI as a priority area for scientific studies and includes a definition on its website (see https://www.nidcd.nih.gov/health/voice/pages/specific- language-impairment.aspx). This shift in perspective was accomplished by dedicated scientists meeting high empirical standards of validity and replication across studies, evaluating well-motivated and competing theoretical interpretations, carrying out population-based epidemiological studies as well as decades-long longitudinal studies, and introducing genetic and family-based programmes of investigation. In effect, a significant knowledge base has accumulated and provides a foundation for further advances (Leonard 1998).

Let me highlight a few noteworthy advances, with selective, not exhaustive, references:

- Valid estimates of population prevalence in young children reveal a relatively high rate, about 7%, of children with SLI (Tomblin *et al.* 1997).
- For children identified with SLI in a population-based study, speech disorders appear in approximately 5–8% of the children, a much lower overlap than expected (Shriberg *et al.* 1999).
- The relationship between language impairments and nonverbal cognitive abilities is not straightforward—nonverbal cognitive deficits are neither necessary nor sufficient for language impairments (Rice *et al.* 2004, Tomblin *et al.* 1997).
- Empirical advances include new methods of language assessment that meet high standards of sensitivity and specificity (Rice and Wexler 2001, Spaulding *et al.* 2006).
- Language impairments of children with SLI are likely to persist throughout childhood and adolescence (Conti-Ramsden *et al.* 2012, Johnson *et al.* 1999, Rice 2013).
- The grammatical property of finiteness marking in English and other languages is a linguistic requirement likely to pose problems for children with SLI, a finding that informs theories of children's language acquisition and has led to ways to identify children with SLI (Rice 2000, Rice and Wexler 1996), develop theoretically coherent empirical measures of progress toward the adult grammar from toddlers to adolescents (Hadley and Holt 2006, Hadley *et al.* 2014, Rice 2012, 2013, Rispoli *et al.* 2012), differentiate dialectal differences from language disorders (Oetting and McDonald 2001), differentiate children with SLI from children with ADHD (Redmond *et al.* 2011), differentiate bilingualism from SLI (Paradis *et al.* 2008), and identify remarkable strengths as well as limitations in growth trajectories across a very wide age range (causing us to rethink our notions of ‘impairment’) (Rice 2012, 2013).
- Significant advances in our understanding of causal pathways, with illumination of previously unknown genetic influences as well as clarification of prenatal, perinatal, and environmental contributions (Rice *et al.* 2008, 2009, 2014).

With these remarkable accomplishments in a relatively short time, why are we now debating the value of SLI as a diagnostic category? The dialogue is inspired in part by the editorial decisions of the recent revision of the Diagnostic and Statistical Manual of Mental Disorders (DSM-5) (2013), compiled by the American Psychiatric Association. DSM-5 serves as an official nosology for mental disorders, meant to be implemented by physicians/clinicians in medical settings. Prominent in the development of the DSM-5 were physicians, psychologists, social workers, nurses, counsellors, epidemiologists, statisticians, neuroscientists and neuropsychologists. The point here is that the vetting process involves diverse professional perspectives.

Communication disorders were considered in the context of neurodevelopmental disorders, including intellectual disabilities, autism spectrum disorder, attention deficit hyperactivity disorder, specific learning disorder and motor disorders. Because the category of SLI met rigorous scientific standards after more than a year of internal editorial reviews, it was included in the nosology that proceeded to the phase of public comments, where it received mixed reviews but not as heatedly mixed as for other proposed categories. As it turned out, the diagnostic language categories of DSM-5 were relevant to controversial changes in the diagnostic category of autism spectrum disorders. Ultimately, a new category of social communication disorder (SCD) was coined, for which, as Bishop observes, reliable and valid assessments are lacking. In short, the empirically well-researched category of SLI was not included and the newly coined category of SCD, with a minimal research base, was included.

My conclusion is that the DSM-5 outcome does not constitute reason to step away from the enormous scientific gains accrued from studies of SLI as currently defined, especially in a world where, as Bishop notes, the term has generated almost 20 000 citations in the scientific literature, an impact that will require a long time for an alternative label to achieve. In the meantime, children (and families) in need of identification remain at high risk of being undetected. One potentially helpful legacy of the DSM-5 is the notion of ‘specifiers’, used, for example, to clarify subgroups within the category, ‘Autism Spectrum Disorder’. For example, one ‘specifier’ is whether ASD appears with or without accompanying language impairment; another is whether ASD appears with or without accompanying intellectual impairment. DSM-5 kept the broadly inclusive term ‘Language Disorders’ (LD) without specifiers. One potentially valuable approach would be to consider SLI as a ‘specifier’ or subgroup of children identified with language disorders, an approach in need of thoughtful consideration. Another lesson from the DSM-5 experience is the need to recognize that among the barriers to utilization of this diagnostic category is the need for cost-effective, time-efficient screening tools for SLI that can be used in doctors’ and psychologists’ offices, large population-based epidemiological or genetic studies, and in the daily activities of school-based practitioners where there are many cost-related pressures to reduce the number of children identified in need of services. Some progress has been made, in the form of 10-min grammar assessments in the relatively narrow age range of 3–9 years (Rice and Wexler 2001), now available free online (see http://www2.ku.edu/~cldp/MabelRice/) and found to show high heritability in twin studies (Bishop *et al.* 2005). Yet much more is needed. Ultimately, parental access to screening tools could be a key asset for breaking down the barriers to access to clinical services.

All things considered, the risk of advocacy for SLI seems very small compared with the risk of derailing a very productive line of scientific enquiry with high relevance for clinical services and the likelihood that, without the label, services for individuals with SLI will be reduced even further.

In the interest of full disclosure, I served as an advisor to the Neurodevelopmental Disorders Work Group for DSM-5, as reported in the manual. In that capacity I worked on a panel charged with the development of the categories for communication disorders. Advisors signed confidentiality agreements as part of the process.

# Other: The SLI construct is a crucial link to the past and a bridge to the future

**Published:** 2014-08-20

**Authors:** Catherine L Taylor

**Affiliations:** Telethon Kids Institute, University of Western AustraliaPerth, WA, Australia

## Abstract

Language impairment constructs are not the province of one discipline, profession, political portfolio, service system or programme of research. What brings different perspectives together is the shared purpose of improving language outcomes and life choices and opportunities for children with language impairments. We will need to define and measure language impairments differently for different purposes and in ways that foster the exchange of knowledge.

## Main text

It is paramount that we have agreed taxonomies and nomenclature to describe children with language impairments. It is clear from the issues that Bishop discusses that this will not be a trivial exercise. She invites us to think about the evidence and counter-evidence for current language impairment terms and constructs, with a main focus on SLI. This is a valuable exercise for proponents and opponents of the SLI construct. Bishop is not opposed to the term ‘specific language impairment’ (SLI) or the SLI construct and points out that it is a widely used term even though it is not understood well enough. SLI describes a prevalent developmental disability that is under-identified in the general population. Changing the term ‘SLI’ is unlikely to resolve this issue, which is one of measurement rather than nomenclature.

Bishop lays out the measurement challenges for us very clearly. Not the least of these challenges is penetrating conventional disciplinary boundaries and integrating across them. She provides an excellent example of the divide between health and education, that unless bridged, leaves children with SLI under-identified and under-serviced. In Australia, the Australian Early Development Index (AEDI) is a new and potentially powerful lever for bridging this divide. Introduced nationwide in 2009, the AEDI is a population-wide measure of child development in the first year of formal school. It covers five developmental domains: physical health and well-being, social competence, emotional maturity, language and cognitive skills, and communication skills and general knowledge. It is completed by a teacher on individual children, but the data are not used to identify individuals. Rather, the data are aggregated at the community level to show the proportion of children who are developmentally on track, at risk and vulnerable. This is a radically different approach to monitoring children's development through screening for clinical assessment and clinical services. The AEDI is not a substitute for speech pathology assessment and speech pathology services: it has a different purpose. The speech pathology profession has an increasing public health role and this will require us to embrace different ways of thinking about children with vulnerable language development. Our clinical services are overstretched and social gradients in access to these services mean that the families who need the services most are statistically least likely to access them. AEDI results for vulnerable language and cognitive skills revealed a clear social gradient, with the highest proportion (18.3%) of vulnerable children living in the most disadvantaged communities and the smallest proportion of vulnerable children (5.4%) living in the least disadvantaged communities. However, extrapolated to the entire Australian population of 250 000 five-year-olds, this equates to 9150 vulnerable children living in communities in the bottom (i.e., most disadvantaged) quintile of socio-economic area disadvantage and 17 350 vulnerable children spread across the other four quintiles of socio-economic area disadvantage. These and other data support a proportionate universalism approach to policies and programmes for children with vulnerable language and cognitive skills (Christensen *et al.* 2014, Taylor *et al.* 2013a). Proportionate universalism is provision of services for all, delivered on a scale and with an intensity proportionate to the level of disadvantage (Marmot 2010). The logic, as illustrated in the AEDI example, is that policies and programmes targeting only the most disadvantaged groups will miss large numbers of vulnerable children.

Advances in knowledge about human development have widened the lens through which we view individual differences in children's language development. Invariably, consideration of the full range of individual differences in children's language abilities makes it difficult to draw boundaries between typical and atypical development. This challenge is not confined to childhood and Bishop draws lessons from chronic diseases such as hypertension and obesity to illustrate this. Perhaps the greatest challenge lies in the temporal dimension of health. As contemporary definitions of health evolve to include health trajectories and transitions, the boundaries between health and disease are becoming even less clear-cut.

Contemporary definitions of language impairment will also need to describe patterns of stability, change, improvement and decline in children's language development. Increasingly childhood is front and centre of public health policy (Taylor *et al.* 2013b). Bishop encourages us to think beyond the language domain to the overarching goal of childhood which is to develop human capability, defined as our capacity to participate economically, socially and civically and to live lives we value (Zubrick *et al.* 2009). The new DisabilityCare Australia national insurance scheme emphasizes the importance of a person's abilities, interests, goals and aspirations in the provision of support services for people with disabilities (Disabilitycare Australia 2013).

Bishop poses the question of whether to keep the term SLI or to change it. From her article we know that the definition of SLI is not fixed and that it has evolved with the science. She illustrates this using the example of how the criterion for nonverbal ability has changed from an outmoded discrepancy criterion (i.e., language ability substantially less than nonverbal ability) to the criterion that nonverbal ability that is broadly within the normal range. She points out that the use of the term ‘specific’ to mean ‘exclusive’ is at odds with the evidence that SLI can co-occur with developmental vulnerabilities in other domains (e.g., social–emotional development). Rather than changing the term SLI, the definition can be updated to include children whose most conspicuous, but not their only, developmental difference is in the language domain. This does mean that the term ‘specific language impairment’ cannot be interpreted literally; however, this is also true for other developmental impairments, such as cerebral palsy. Perhaps, somewhat surprisingly, cerebral palsy is a qualitative trait beset with similar definitional issues to SLI. Like SLI, it is an umbrella term for a heterogeneous group of impairments, in this case motor impairments, with largely uncharted aetiologies. The risk factors for SLI, and most cases of cerebral palsy, are essentially unremarkable, in that most children with these risk factors (i.e., preterm birth, intrauterine growth restriction) have normal development. As we confront definitional challenges in relation to language impairments, we can learn from how these challenges are being met in relation to other developmental disorders. For example, the term ‘cerebral palsy’, while not perfect, has been kept because it provides an important link to the past. Like SLI, the definition of cerebral palsy has been updated as new evidence has emerged (Stanley *et al.* 2000).

On balance, there is a strong argument for keeping the term SLI (Rice 2009), and as Bishop suggests, updating the definition as our knowledge advances. Keeping the term SLI maintains our link to the past and is a bridge to the future. It is paramount that children, whose most conspicuous individual difference is in the domain of language, are conspicuous in our policies and practice. Research reported in this publication was supported by the National Institute On Deafness and Other Communication Disorders of the National Institutes of Health under Award Number R01DC005226. The content is solely the responsibility of the authors and does not necessarily represent the official views of the National Institutes of Health.

# Other: What should we call children who struggle to talk? Taking a developmental, global perspective on diagnostic labels—reflections on Bishop

**Published:** 2014-08-20

**Authors:** Gina Conti-Ramsden

**Affiliations:** School of Psychological Sciences, University of ManchesterManchester, UK

## Abstract

This commentary takes four alternative diagnostic labels discussed by Bishop and highlights issues that arise from each of the choices. It reflects on the need to have a global, developmental perspective in tackling complex issues of diagnosis and terminology and in moving the field forward.

## Main text

Language learning can be challenging for some children at different times in their lives. Despite the recognized importance of language to children's healthy development, professionals and academics working in speech and language therapy, psychology and education have struggled to find a common language to refer to these children. Currently, we do not have a label that fosters information exchange and collaboration across disciplines and across different stages of children's development. But there is more to this situation. As professionals and academics, we find ourselves dissatisfied and in turmoil: there is terminological confusion, a long list of different labels and different definitions. In terms of diagnosis and terminology we are in the eye of the storm.

Thus, Bishop's article is a particularly welcomed, timely contribution. It provides a very useful, insightful and systematic review of the issues. Bishop not only maps out where we are at but also how we may move forward. In terms of diagnostic labels, in essence, she puts four options for consideration:

- Retain specific language impairment (SLI) and adopt laxer criteria.
- Adopt the diagnostic label PLI.
- Adopt the diagnostic label developmental language disorders (DLD).
- Adopt the diagnostic label language learning impairments (LLI).

Bishop presents a considered discussion of her proposed diagnostic label alternatives. What other considerations may we take into account? In terms of the diagnostic label SLI there are further issues that counter the benefits of retention. First, it is difficult to foster change of criteria for an existing diagnostic label (SLI) particularly when another label is in use and has already taken that semantic space, so to speak. I am referring to the diagnostic label of language impairment (LI) as there are studies that specifically differentiate SLI from LI on the basis of performance IQ criteria being more lax for LI (Weismer *et al.* 2000). Second, Bishop is right in pointing out that SLI is by far the most common label used in academic settings. However, I would like to add the proviso ‘in English’. An examination of number of returns for terms on Google Scholar search for Spanish labels suggests a different picture. In Spanish LI (Spanish: *Trastornos del Lenguaje*) is by far the most common label with 68 000 hits, LLI (*Trastornos del Aprendizaje del Lenguaje*) is next with 36 000 hits; and SLI (*Trastornos Específicos del Lenguaje*) produces 16 000 hits. Taking into consideration professionals and academics working with languages other than English affords a more global perspective on the terminological and diagnostic issues we are facing.

There is also the issue of labels that highlight a particular feature, e.g. ‘primary’ or ‘developmental’. The difficulties with the qualifier ‘specific’ have been well rehearsed so I will not repeat them here. But how about the qualifier ‘primary’? I don't think the change from specific to primary gets rid of enough unwarranted implications. The term ‘primary language impairment’ does not foster a global, developmental way forward. By global this time I mean taking into consideration the perspectives of other disciplines and services.

Take Bishop's case of 8-year-old George. This time George had his first point of contact with clinical psychological services given his temper tantrums as a young pre-schooler, his lack of regular sleeping patterns and his aggression towards his baby sister. The psychologist in consultation with the psychiatrist has diagnosed him as having emotional behavioural difficulties (EBD). At school entry his teacher notices he has a weak vocabulary and he seems to have a hard time following verbal instructions. So she asks her speech and language therapy colleague to undertake an assessment. The diagnosis comes back: George has a primary language impairment. This vignette illustrates some further tensions that may be worth considering. Should we infer that George's primary problem is his language and his EBD is secondary to this previously unidentified difficulty? If the evidence suggests George has co-morbid PLI and EBD, what is the use of the ‘primary’ in the PLI label? One could say that we are identifying a language impairment that is not accounted for by bilingualism so the term PLI is still informative. Would we change our minds if I told you George's mother speaks only Spanish in the home although her English is virtually native-like?

The term ‘primary language impairment’ may also invite a static view of language difficulties across development. Do adolescents who have received speech therapy throughout their childhood and elementary schooling who struggle to understand long, complex sentences in everyday rapid conversations with peers have a primary language problem at this stage in their development? Evidence from the Manchester Language Study would suggest this is not the case, hence our terminology ‘with a history of SLI’ which, in all frankness, is a mouthful (Conti-Ramsden *et al.* 2012).

Leaving aside the issues of the term ‘disorder’ that, as Bishop suggests, could be replaced with ‘impairment’, how about the label developmental language disorder? In this label we have ‘developmental’ clearly highlighted. But does ‘developmental’ here mean what we have been talking about above? Bishop underlines the difficulty of the same term having different meanings in our field and this I would argue is one of the drawbacks for the diagnostic label developmental language disorder. In this context, the term ‘developmental’ is usually interpreted as ‘not acquired’ and ‘in childhood’. This has consequences for young people with language impairment in adolescence and young adulthood. Indeed, one of the challenges in our field is the provision of language support for secondary school pupils and for post-school-age young people. They are at risk of falling off the radar from childhood and receiving child services to their official classification as ‘adults’ and being eligible to access adult services.

Where does this leave us? On the one hand we have the proposal from Bishop for LLI and the proposal from Reilly *et al.* (2014) for LI. Each has its merits that have been described by the authors. I will not add to these but instead underline the need for due process in undertaking further discussions to reach agreement. I would join Reilly and colleagues in calling for an International Consensus Panel; an international and global panel that includes voices from different languages, and the voices of those affected and their families. I would add to the remit of the panel and all those working with language impairment Bishop's call for enhancing positive aspects of development. We need to discuss the inclusion of different areas of functioning in our assessments. We require this information in order to identify the strengths of children and young people with language (learning) impairments. In this respect, we could take a page from the field of autism. If asked, academics and professionals working with autism can tell us what are the most common strengths (Baron-Cohen and Belmonte 2005). Could we answer this question now, today for SLI?

We cannot go on with the current situation. Our mission is much more than reaching agreement on an appropriate label. We are ready to have an open discussion and harness existing goodwill and energy and not only tackle complex issues of diagnosis and terminology but also move the field forward. We can all live with what Bishop and others have highlighted: labels have a necessary place in diagnostic evaluation, but they never tell the whole story.

By a name I know not how to tell thee who I am.

(William Shakespeare, *Romeo and Juliet*, Act 2, Scene 2)

# Other: Changing labels for a concept in change

**Published:** 2014-08-20

**Authors:** Kristina Hansson, Olof Sandgren, Birgitta Sahlén

**Affiliations:** Department of Logopedics, Phoniatrics, and Audiology, Lund UniversityLund, Sweden

## Abstract

In this commentary we reflect on current labels and criteria for child language impairment from a Swedish perspective. We call for a new label highlighting the developmental, changeable and dynamic characteristics of the impairment and discuss implications for diagnosis, assessment and research. Conceptual coherence will promote the professional identity and status necessary for much needed communicability.

## Main text

First of all, this initiative is laudable and we hope that this issue of the journal will mark an important point within research and clinical management of child language impairments. The arguments for diagnosing are strong and we need a research label to match the diagnosis.

Descriptions of a child's strengths and weaknesses and/or an identification of their ‘needs’ are not sufficient but provide the necessary foundation for the diagnosis. We agree with Bishop that the lack of explanation of the problems of children with language impairment is not a reason for not having a label. Labels and diagnoses facilitate communication with other professionals, families and policy-makers but require acknowledgment of the large amount of heterogeneity that is characteristic of all neurodevelopmental conditions. Within the field of SLI this raises the question of the specificity of the impairment, an issue that has for a long time caused debate among child language researchers in our country. The strong influence of Stark and Tallal (1981) has been challenged by almost 35 years’ of intense exploration of the neurobiological, genetic and social underpinnings of child language impairments. This has altered the picture and today we prefer the label ‘language impairment’, which, in our view, shows potential to better unite research and clinical interests.

Language impairment is elusive in the sense that the constellation of strengths and weaknesses within a child is not static but changes with both time and context. We cannot predict what problems or skills will persist or even emerge. We need to remind ourselves that language processing involves interaction in two senses: an interaction between cognition, language and sensorimotor systems within the individual as well as between individuals involved in interpersonal communication. A deficient or reduced functioning in any of these systems may result in limitations in communicative choices and use of compensatory strategies. This complex interaction of systems within and between individuals generates unique effects for each individual and for each communicative event. This also opens up the possibility for the emergence of new skills and abilities through compensatory adaptation and choices (Perkins 2007). The highly contextual nature of language processing calls into question our standardized assessment procedures. The influence of non-verbal aspects of the communicative setting, e.g. the partner's gaze, gestures and voice characteristics, are often overlooked. We have recently shown that both the examiner's speech rate and voice quality interact in interesting ways with item complexity and cognitive factors to affect children's performance in a language comprehension test (Haake *et al.* 2014, Lyberg-Åhlander *et al.* 2014). Applying the framework of the International Classification of Functioning, Disability and Health (ICF) when deciding on a new label to be used for both research and clinical purposes would be useful. This will give clinicians a better understanding of the functional consequences for the affected child and will help determine appropriate goals for intervention and research (McLeod and Bleile 2004).

Similarly to colleagues in English-speaking countries, diagnoses available for Swedish clinicians, described within the ICD-10, do not map neatly onto the research label. Whereas SLI gives the impression of enabling clear-cut distinctions between affected and unaffected children, clinicians struggle to find similar delineations. This results in frustration from researchers hoping to recruit participants, and frustration from clinicians turning to research in search of answers to manage their everyday caseloads. Due to lack of alternatives, clinicians agree on less than optimal diagnostic codes, with F80.2B (mixed receptive–expressive language disorder) providing the closest match for the research label SLI.

As for the question of cognitive referencing, most research in our country (articles published during the last 10 years) apply a cut-off for non-verbal IQ at 70. This cut-off point has never been officially discussed in the research community, only gradually adopted by researchers.

Support for the convention is provided by results showing similar benefits of intervention for children with language impairment with IQ between 70 and 80 and those above 80 (e.g., Tomblin 2008).

In Sweden, phonological difficulties have been considered part of language impairment since the early 1980s (Nettelbladt 1983). At least at pre-school age, children with grammatical/lexical problems almost invariably also present with some degree and type of phonological difficulties. There seems to be less agreement on including social communicative problems in language impairment. According to our experience, individuals with such deficits emerging from structural language problems (i.e., problems at different levels of language production and comprehension) qualify for inclusion even with apparently restored language abilities. Thus, it is our opinion that children who have not had any structural problems should not be included.

A growing body of research points to co-occurrence of language impairment and other conditions. In our own research comparing children with mild to moderate sensorineural hearing impairment and children with language impairment (e.g., Sahlén and Hansson 2006, Sandgren *et al.* 2013) the findings are consistent with those of other authors (e.g., Briscoe *et al.* 2001, Gilbertson and Kamhi 1995). A considerable proportion of children with hearing impairment have similar problems as children with language impairment, but their problems are generally not as pervasive and persistent. The lack of proportionality between degree of hearing impairment and degree of language deficits suggests that hearing and language impairments co-occur.

We should recognize and take pride in the contributions of SLI theory to the research on language impairments in a range of disability groups. Although much remains to be explained, the development of SLI theory has informed us on both typical and atypical language development, and how language relates to and interacts with other cognitive and social skills. The urge to increase the theoretical and methodological depth should not prevent us from taking a stance on the label and diagnostic criteria and to advance the field.

The time has come for a new label and a golden standard for definition, criteria and assessment of children with language impairment. Conceptual coherence will enable and facilitate communication between researchers, clinicians, families, educational systems and policy-makers. This will strengthen the identity of researchers and clinicians working in the field of child language disorders. This, in turn, will better the chances of communicating the message. In our opinion, language learning impairment and developmental language disorder are both likely to accomplish these objectives since the labels highlight the dynamic, changeable nature of the condition.

# Other: What should we call children with unexplained language difficulties? A practical perspective

**Published:** 2014-08-20

**Authors:** Ann Clark, Glenn Carter

**Affiliations:** * Queen Margaret UniversityMusselburgh, UK; † NHS Forth ValleyUK

## Abstract

This commentary reflects on Bishop's discussion of possible diagnostic terms for children with unexplained language problems. We discuss each of her four proposed terms in turn, commenting on their potential use in clinical and educational contexts by speech and language therapists and other professionals.

## Main text

Bishop's article is a timely and welcome discussion, gathering together the main issues around the terminology used to describe children who have unexplained language problems.

There are many UK-wide examples of excellent SLT practice working with children with unexplained language problems in increasingly challenging professional environments, often with reduced resources. Although SLTs appear to have a consistent approach to interventions with children with unexplained language problems, there is no consistent practice either in what we call these children or in the pathways to their diagnosis. In our survey of SLTs working in Scotland (Clark *et al.* 2013), we found 85% used ‘specific language impairment’ in line with Bishop's finding with Google Scholar. However, although SLI is the term used most often by SLTs, 45% also used ‘language disorder’, 27% ‘language delay’ and 21% ‘specific language disorder’. This adds further weight to Bishop's view that the current system is in mayhem. Some SLTs report they have moved away from the term SLI when discussing these difficulties with parents and education colleagues and use it only with SLT colleagues.

In terms of pathways to diagnosis, we have increasingly moved away from traditional exclusionary criteria and towards measuring and defining inclusionary criteria that best represent difficulties these children present with. In the survey, 6% said diagnosis was a joint process between SLTs and educational psychologists, 12% said this took place between SLTs and specialist language teachers. However, 82% said there was either no official pathway of diagnosis (59%) or they did not know/were not sure what it was (23%). Despite the fact, as Bishop discusses, that cognitive referencing is now largely discredited, this information can be slow to disseminate onto the ground. SLTs may be reluctant to change their practice because there has been no clear message about alternative and evidence-based pathways in which to make a diagnosis. However, one thing is clear. SLTs have a key role in diagnosis and we need sharper tools to achieve this, while working with education colleagues to identify the impact for each child.

Bishop presents a vignette to illustrate her arguments. At present it is difficult to give George a diagnosis that is consistently understood or valued by the team around him including his parents. The lack of a consistent, known label is likely to lead to confusion and indeed anger for George's parents who are trying to come to terms with his difficulties, but are unable to qualify information with a consistent message from professionals and the internet. The internet is the most powerful tool parents, clinicians and young people have for accessing information that can inform and help them understand the nature of these difficulties. Without agreement on a label, accessing this information is frustrating or worse misleading. One of the positive consequences of diagnostic labels, to which Bishop alludes, warrants further comment. That is, the fact that a ‘reason’ or label for a child's difficulties can help to ‘remove the blame from the child’. This will be particularly salient for George as he gets older. Anecdotal evidence and experience working with teenagers and adults shows that they feel disempowered by not understanding the nature of their difficulty and believe the feedback from their peers or others that they are ‘stupid’ or ‘useless’. A clear diagnosis and label would help to reduce George's anxiety.

Bishop presents four terms as potential candidates to describe unexplained language problems, economically and persuasively outlining their pros and cons, as well as possible ways forward. Here, we hope to add to the debate by discussing some other issues around these four suggestions in the context of SLT practice.

With respect to SLI, the fact that there are so many different labels used in the literature and in practice suggests that this term has not met the mark. One suggestion is to change the understood meaning of ‘specific’ to ‘idiopathic’. While keeping the term SLI is appealing, as it is so widely used it would be challenging, and arguably it is too late, to redefine a term which, for so long, has meant something different.

Another challenge is differentiating between children who may have transient language difficulties and those who have an atypical/disordered language profile. Transient language difficulties is a term that well describes children with delayed language presentation that responds to generalized language interventions such as vocabulary development or narrative techniques. One possibility would be to hold onto SLI for the small group of children who actually have very specific difficulties and further build on the term ‘transient language difficulties’ for children who have delayed language difficulties related to environmental deprivation, for example. We would only know which group a child falls into after we have seen how they respond to intervention. Reilly *et al.* (2014) recommend waiting a minimum of a year before making a diagnosis, which seems a sensible guideline.

Moving on to the term ‘primary language impairment’, ‘primary’ has the advantage of narrowing down ‘language impairment’ while neatly avoiding the problems of ‘specific’. However, ‘primary’ could lead to confusion with parents and education staff as it potentially implies these difficulties are relevant to children who are of ‘primary’ school age. It excludes the reality that that this is a developmental difficulty, which for the majority of affected children first manifests in the early years and in many cases continues into adolescence.

‘Language learning impairment’ is the most education friendly term suggested by Bishop. A benefit of this term is that it ties ‘language’ with ‘learning’ to emphasize the impact of these difficulties on educational success. One potential problem is that ‘learning’ may suggest that difficulties are associated with formal learning at school only, rather than language learning in other contexts as well.

Considering the term ‘developmental language disorder’, the addition ‘developmental’ to the DSM-V term ‘Language Disorder’ is helpful, as it focuses on the congenital aspect of these difficulties. Within the context of education colleagues it is still useful within the early and primary school years. It may be more problematic as adolescence approaches. However, adults with ‘developmental dyslexia’ diagnosis commonly drop the ‘developmental’ and we perhaps need to take a pragmatic approach in adopting a term that reflects at what stage we most commonly provide support.

When we weigh up Bishop's arguments about the positive and negative consequences of a consistent label, we are in absolute agreement with Bishop's statement that ‘labels can have negative consequences, but the consequence of avoiding labels can we worse’.

Where do we go from here? We need an urgent, strategic and international debate to come to an informed and ideally a consensus decision on what term we adopt. It needs to be precise enough to describe the difficulties these children face and understandable to those outside of our profession. However, that said, no matter what we call it, we need to use the term to increase awareness of the difficulties faced by these children. If we can do that and the profile is high enough, we can help the general population attach meaning to a particular term, as we have seen with autism spectrum disorder. Then we will be in a much stronger position to campaign for resources to support these children and for research funding to increase our knowledge of both underlying causes and effective interventions. The adopted term needs to be integrated into SLT pre- and post-registration training, as well into training of education staff including educational psychologists.

This debate is timely, needed and welcome. Let us continue with the momentum we now have and aim to ensure we will not be having the same discussion 10 years from now.

# Other: Terminology mayhem: why it matters—the ramifications for parents and families

**Published:** 2014-08-20

**Authors:** Alison Hüneke, Linda Lascelles

**Affiliations:** Afasic, UK

## Abstract

Parents take the view that the term ‘SLI’ has not been a successful diagnostic label. Its lack of credibility and market recognition has made it vulnerable to political and economic pressures. Parents would welcome a term that helps ensure their children's difficulties are recognized, taken seriously and adequately supported. Alternatives are briefly considered and improvements suggested.

## Main text

Afasic is the UK charity representing parents of children who might be described as having SLI as well as other forms of speech and language difficulty. As such, we are well placed to reflect parents’ views about diagnostic terms.

Any debate about SLI must take account of the prevailing political and social context. Until comparatively recently, SLI was generally regarded as a relatively rare condition requiring a high level of specialist intervention; however in recent years there has been more emphasis on the need for a ‘mass intervention’ approach to address the substantial minority of children now recognized to have what has more frequently come to be called ‘speech language and communication needs’ (SLCN).

Why this shift in how language difficulties are perceived? Firstly, the focus on underachievement among young people from low socioeconomic backgrounds has identified speech and language difficulties in the early years as one of the main causative factors. Secondly, financial constraints imposed on the NHS have meant considerable cuts to speech and language therapy services, including the loss of many specialist therapists, and a shift away from personalized therapy towards generalized, low-dosage packages of support, often delivered by early years or support staff.

However, the use of ordinary English words in terms such as SLI or language delay/disorder has probably not helped either. It is hard to imagine such a dramatic change happening to a more ‘medical’ sounding label, such as autism or ADHD. Even some medical professionals are struggling. Paediatricians, for instance, increasingly seem to consider SLI to be an ‘educational’ issue rather than a medical condition.

Another issue is that the underlying concept does not really resonate. We seem to be hardwired to assume that young children will learn to talk in due course, and there do not seem to be enough children with visibly serious but unexplained problems to change this perception.

Clearly, then, SLI has not been a very successful term. Both Bishop and Reilly *et al.* (2014) argue that it is not helpful to retain it as it is currently understood. Their view is that SLI cannot be distinguished clearly from non-specific language impairment, which they suggest has in the past been regarded as less serious. They argue that, in fact, children with low non-verbal IQ respond equally well to speech and language therapy and have similarly poor, or in some cases even worse, adult outcomes. Bishop also adds that the term ‘language disorder’ can be misleading as it may be interpreted as a more severe condition than a broader delay whereas the evidence points to a much better prognosis.

Parents would argue that this is precisely the point. They are happy to concede that, compared with many other disabilities, their child's difficulty is relatively mild. The difference is that, given the right support, these children have the potential to do as well as their non-impaired peers, achieving good GCSEs, securing meaningful employment and even going on to university.

The authors of the lead articles in this special issue may take the view that removing the distinction between specific and non-specific language impairment will mean that both groups of children receive the higher level of help that parents of children with SLI currently (sometimes) secure. This is optimistic. In the current climate, where both education and health services are reluctant to fund speech and language therapy, there is a high risk that they will instead offer only a very basic level of support to everyone, with the result that the concept of language impairment as a specific need will effectively disappear, and instead be subsumed into general low ability/mild learning difficulty.

Nevertheless, it is possible that all these concerns could be addressed by the adoption of a more viable alternative label. Bishop does suggest a number of possibilities, none of which is likely to be any more successful than SLI has been. Of the various options, parents would probably favour the term ‘disorder’, which, they feel, indicates a specific problem. We recently consulted a small group of parents about the term ‘language learning impairment’, but they felt it implied a learning difficulty and would be equated with low ability.

The best option would be something like ‘(developmental) dysphasia’: it is clearly a medical term; it equates SLI with other specific learning difficulties such as dyslexia and dyspraxia; and it meets the ‘Google test’ outlined by Bishop. It is also the standard term in other European countries, including France.

An alternative option, as Bishop suggests, is to revise the definition of language impairment. Criteria that depend on language scores require children to have a full speech and language therapy assessment, and only a very small number do so. Classifying it instead in terms of specific abnormalities in children's language would have a number of advantages:

- It would be easier for teachers and other non-specialists to recognize.
- It could also be applied to brighter children who may be struggling with some aspect of comprehension or expressive language, but whose scores are too high to meet current criteria. Their inclusion would also, as with dyslexia, help to shift the perception of the condition more positively.
- It would make it easier to identify children with social communication disorders who often score quite well on formal language assessments and so struggle to have their needs recognized and met.
- The identifying characteristics could be adapted for a range of ages. This would help to remove the perception that language deficits relate solely to a difficulty with the basic language skills young children normally acquire during their pre-school years and raise awareness that they can affect people of any age.

Initially, parents often worry that having a label might adversely affect their children as they grow up, but they do welcome something that helps them understand their children's difficulties and validates their concerns. What they would like, above all, is a diagnostic label that other people recognize and understand, and which delivers the help their children need.

# Other: Getting behind the label: practitioners’ points of view

**Published:** 2014-08-20

**Authors:** Marion Strudwick, Ann Bauer

**Affiliations:** * Parent advocate SOS!SEN, the special needs helpline; † Head of Language Resource, mainstream secondary school

## Abstract

Dorothy Bishop's article concerning the labelling of language impairments is discussed in the context of educational provision. We discuss labelling from our perspectives as parent advocate and specialist teacher. We support the need for labels and for a more universal definition of terms. We suggest the label becomes a starting point to describe the child's language needs within a holistic profile.

## Main text

We welcome the debate on precise labelling of speech and language needs. Labels have a use as they provide a reference to need, in this case in relation to speech and language, and also a foundation for provision, ‘we could then gather evidence to determine which children actually benefit from support and services’ (Bishop). They are also a basis upon which specialist teaching, therapy and mainstream teaching can develop (McCartney *et al.* 2009: 80–90). The name attached to this need is significant with Bishop explaining the possible impacts on pupils’ education arising from attitudes towards the condition. Reilly *et al.* go further in suggesting cut-off data to form criteria for inclusion into whatever label is chosen. We hesitate about the use of cut-off data and argue that the child's language needs should be considered in the context of the whole child's profile.

‘Language impairment’ (LI) (Reilly *et al.* 2014) or ‘language learning impairment’ (LLI) (Bishop) are both acceptable titles and we welcome Bishop's warning that the label is not the construct. We contend that labels are open to misunderstanding and misinterpretation by some professionals. In our experience, misinterpretation leads to inappropriate provision for an individual child, whether determined within school, local authority or special educational needs tribunal. Therefore, any universal label should have enough depth to be understood by all.

We have experienced the difficulties facing those determining a child's provision to understand the implications and differences in terminology relating to impairments described variously as ‘delayed’, ‘disordered’, ‘difficulties’, ‘needs’ (Bishop). For instance, a teacher describing all pupils as having ‘individual needs’ gives this as a reason why a pupil's language ‘needs’, described in a statement of SEN, do not need to be addressed differently, or a tribunal member claiming all speech and language therapists have their own interpretation of what is disordered and what is delayed. This can have serious consequences for the outcomes for the child (Bishop) if it leads to failure to address the impairment.

We suggest that either LI or LLI are valid labels, but if a child's needs are to be fully understood and met, the label is just the starting point. The label is not necessarily helpful unless two things are addressed: (1) the nature of the impairment(s); and (2) the whole profile of the child covering the range of needs, how they impact upon each other and the holistic outcome. Bishop shows there are very few pupils who have language impairment as their only special educational need.

We contend that the broad label ‘language impairment’ is only useful if the nature of the impairment is clearly set out to indicate whether the language is delayed, disordered or both, whether it relates to fundamentals such as pragmatics, semantics, auditory processing, short-term memory or combinations of difficulties. The language impairment should also be set out in the context of the child's developmental history as well as other defined needs, for instance cognition, attainment, emotional and sensory. Billington (2000: 111) cites Donna Williams’ description of her own emotional shutdown when sensory overload became too stressful.

The difficulty of a label without qualification is that a pupil with co-morbid needs may have one need addressed at the expense of another, without considering the impact of one upon another, e.g. a pupil with language impairment may also have an autism spectrum condition (ASC) and dyslexia. In this case the pupil *may* require an integrated approach using a separate table, privacy screen, scaffolded support for work to be undertaken, including vocabulary cues, and access to a laptop and dyslexia-friendly programmes such as ‘Texthelp’ in order to succeed at a given task. If this pupil was labelled primarily as having language impairment, or dyslexia (another contentious term), or ASC then the pupil may not be given a suitably integrated approach to learning, as Reilly *et al.* (2014: 36) state ‘the degree to which the child's LI would be the focus for intervention and the intervention approaches chosen, would vary depending on the individuals’ profile and the holistic needs of the child’. We have to be careful to avoid fragmented provision by prioritization.

We suggest that by constructing a holistic picture of the child, of which the language impairment is part, we are more likely to lead to adequate provision being made for the pupil. This avoids the idea of a main presenting need that can avoid meeting the full profile of needs of a child, e.g. the temptation to send a pupil, who on usual standardized testing appears to have moderate learning difficulties (MLD), to what is primarily an MLD school could be inappropriate if that pupil also presents with significant language impairment which may have affected his/her attainment on some cognitive tests.

In conclusion, we support the view that a universally understood term for speech and language impairments/needs/difficulties/delays/disorders is overdue. The variety of language impairments and their presentation either as part of a medical condition, or as a co-morbid condition, or as acquired or inherited suggests that we either choose to develop an inclusionary—with an exclusionary corollary—list of criteria or that we use it as a signifier of details to follow. We argue that any label of language impairment should carry with it a clear language profile of the child in his/her educational and developmental context. The new draft code of practice (2013), which is due to take effect in September 2014, makes the ‘key requirement’ that educational establishments:

**must**:

- use their best endeavours to ensure that the necessary provision is made for any individual who has SEN;

[…] This means provision that goes beyond the differentiated approaches and learning arrangements normally provided. […] It may take the form of additional support from within the setting or require the involvement of specialist staff or support services. (Department for Education (DfE) 2013: 58–59)

‘Necessary provision’ requires clear understanding of need. A label will only be as good as its meaning, otherwise it becomes packaging rather than the contents.

## References

[b94] American Psychiatric Association (2013).

[b95] Baron-Cohen S, Belmonte MK (2005). Autism: a window onto the development of the social and the analytic brain. Annual Review of Neuroscience.

[b96] Billington T (2000). Separating, Losing and Excluding Children.

[b97] Bishop DVM, Adams CV, Norbury CF (2005). Distinct genetic influences on grammar and phonological short-term memory deficits: evidence from 6-year-old twins. Genes, Brain, and Behavior.

[b98] Briscoe J, Bishop DVM, Norbury CF (2001). Phonological processing, language, and literacy: a comparison of children with mild-to-moderate sensorineural hearing loss and those with specific language impairment. Journal of Child Psychology and Psychiatry.

[b99] Christensen D, Zubrick S, Lawrence D, Taylor C (2014). Risk factors for low receptive vocabulary abilities in the preschool and early school years in the Longitudinal Study of Australian Children. PLoS One.

[b100] Clark A (2013). Tangled terminology: what's in a name. Royal College of Speech and Language Therapist's Bulletin.

[b101] Conti-Ramsden G, St Clair MC, Pickles A, Durkin K (2012). Developmental trajectories of verbal and nonverbal skills in individuals with a history of specific language impairment: from childhood to adolescence. Journal of Speech, Language, and Hearing Research.

[b102] Department for Education (DfE) (2013). https://www.education.gov.uk/consultations/downloadableDocs/DraftSENCodeofPractice.pdf.

[b103] Disabilitycare Australia (2013). http://www.ndis.gov.au.

[b104] Elliott JG, Gibbs S (2008). Does dyslexia exist. Journal of Philosophy of Education.

[b105] Gilbertson M, Kamhi AG (1995). Novel word learning in children with hearing impairment. Journal of Speech and Hearing Research.

[b106] Haake M, Hansson K, Schötz S, GULZ A, Sahlén B (2014). The slower the better? Does the speaker's speech rate influence children's performance on a language comprehension test. International Journal of Speech–Language Pathology.

[b107] Hadley PA, Holt J (2006). Individual differences in the onset of tense marking: a growth curve analysis. Journal of Speech, Language, and Hearing Research.

[b108] Hadley PA, Rispoli M, Holt JK, Fitzgerald C, Bahnsen A (2014). The growth of finiteness in the third year of life: replication and predictive validity. Journal of Speech, Language, and Hearing Research.

[b109] Johnson C, Beitchman JH, Young A, Escobar M, Athkinson K, Wilson B, Brownlie EB, Douglas L, Taback N, Lam I, Wang M (1999). Fourteen-year follow-up of children with and without speech/language impairments: speech/language stability and outcomes. Journal of Speech, Language, and Hearing Research.

[b110] Lauchlan F, Boyle C (2007). Is the use of labels helpful in special education. Support for Learning.

[b111] Leonard LB (1981). Facilitating linguistic skills in children with specific language impairment. Applied Psycholinguistics.

[b112] Leonard LB (1991). Specific language impairment as a clinical category. Language Speech and Hearing Services in Schools.

[b113] Leonard LB (1998). Children with Specific Language Impairments.

[b114] Lyberg-Åhlander V, Haake M, Brännström J, Schötz S, Sahlén B (2014). Does the speaker's voice quality influence children's performance on a language comprehension test. International Journal of Speech–Language Pathology.

[b115] Marmot M (2010). http://books.google.co.ug/books?id=AcfESAAACAAJ.

[b116] McCartney E, Ellis S, Boyle J (2009). The mainstream primary classroom as a language-learning environment for children with severe and persistent language impairment—implications of recent language intervention research. Journal of Research in Special Educational Needs.

[b117] McLeod S, Bleile K (2004). The ICF: a framework for setting goals for children with speech impairment. Child Language Teaching and Therapy.

[b118] Nettelbladt U (1983). Developmental Studies of Dysphonology in Children.

[b119] Oetting JB, McDonald JL (2001). Nonmainstream dialect use and specific language impairment. Journal of Speech, Language, and Hearing Research.

[b120] Paradis J, Rice ML, Crago M, Marquis J (2008). The acquisition of tense in English; distinguishing child second language from first language and specific language impairment. Applied Psycholinguistics.

[b121] Perkins M (2007). Pragmatic Impairment.

[b122] Pinker S (1984). Language Learnability and Language Development.

[b123] Redmond SM, Thompson HL, Goldstein S (2011). Psycholinguistic profiling differentiates specific language impairment from typical development and from attention-deficit/hyperactivity disorder. Journal of Speech, Language, and Hearing Research.

[b124] Rees I, Kelly B, Woolfson L, Boyle J (2008). A systemic solution-oriented model. Frameworks for Practice in Educational Psychology.

[b125] Reilly S, Tomblin B, Law J, McKean C, Mensah F, Morgan A, Goldfeld S, Nicholson J, Wake M (2014). Specific language impairment: a convenient label for whom. International Journal of Language and Communication Disorders.

[b126] Rice ML, Bishop DVM, Leonard LB (2000). Grammatical symptoms of specific language impairment. Speech and Language Impairments in Children: Causes, Characteristics, Intervention and Outcome.

[b127] Rice ML, Gathercole VCM (2009). Language acquisition lessons from children with specific language impairment: revisiting the discovery of latent structures. Routes to Language: Studies in Honor of Melissa Bowerman.

[b128] Rice ML (2012). Toward epigenetic and gene regulation models of specific language impairment: looking for links among growth, genes, and impairments. Journal of Neurodevelopmental Disorders.

[b129] Rice ML (2013). Language growth and genetics of specific language impairment. International Journal of Speech–Language Pathology.

[b130] Rice ML, Smith SD, Gayán J (2009). Convergent genetic linkage and associations to language, speech and reading measures in families of probands with specific language impairment. Journal of Neurodevelopmental Disorders.

[b131] Rice ML, Taylor CL, Zubrick SR (2008). Language outcomes of 7-year-old children with or without a history of late language emergence at 24-months. Journal of Speech, Language, and Hearing Research.

[b132] Rice ML, Tomblin JB, Hoffman L, Richman WA, Marquis J (2004). Grammatical tense deficits in children with SLI and nonspecific language impairment: relationships with nonverbal IQ over time. Journal of Speech, Language, and Hearing Research.

[b133] Rice ML, Wexler K (1996). Toward tense as a clinical marker of specific language impairment in English-speaking children. Journal of Speech and Hearing Research.

[b134] Rice ML, Wexler K (2001). Rice/Wexler Test of Early Grammatical Impairment.

[b135] Rice ML, Zubrick SR, Taylor CL, Gayán J, Bontempo DE (2014). Late language emergence in 24 month twins: heritable and increased risk for twins. Journal of Speech, Language, and Hearing Research.

[b136] Rispoli M, Hadley PA, Holt JK (2012). Sequence and system in the acquisition of tense and agreement. Journal of Speech, Language, and Hearing Research.

[b137] Sahlén B, Hansson K (2006). Novel word learning and its relation to working memory and language in children with mild-to-moderate hearing impairment and children with specific language impairment. Journal of Multilingual Communication Disorders.

[b138] Sandgren O, Andersson R, van de Weijer J, Hansson K, Sahlén B (2013). Impact of cognitive and linguistic ability on gaze behavior in children with hearing impairment. Frontiers in Psychology.

[b139] Shriberg LD, Tomblin JB, McSweeny JL (1999). Prevalence of speech delay in 6-year-old children and comorbidity with language impairment. Journal of Speech, Language, and Hearing Research.

[b140] Spaulding TJ, Plante E, Farinella KA (2006). Eligibility criteria for language impairment: is the low end of normal always appropriate. Language, Speech, and Hearing Services in Schools.

[b141] Stanley F, Blair E, Alberman E (2000). Cerebral Palsies: Epidemiology and Causal Pathways.

[b142] Stark RE, Tallal P (1981). Selection of children with specific language deficits. Journal of Speech and Hearing Disorders.

[b143] Taylor C, Christensen D, Lawrence D, Mitrou F, Zubrick S (2013a). Risk factors for children's receptive vocabulary development from four to eight years in the Longitudinal Study of Australian Children. PLoS ONE.

[b144] Taylor C, Zubrick S, Rice M, Rescorla L, Dale P (2013b). Population and public health perspectives on late language emergence at 24 months as a risk indicator for language impairment at 7 years. Late Talkers: Language Development, Interventions, and Outcomes.

[b145] Times Educational Supplement (TES) (2007). Don't read the label, it's damaging. Times Educational Supplement.

[b146] Tomblin JB, Norbury CF, Tomblin JB, Bishop DVM (2008). Validating diagnostic standards for SLI using adolescent outcomes. Understanding Developmental Language Disorders.

[b147] Tomblin JB, Records NL, Buckwalter P, Zhang X, Smith E, O'Brien M (1997). The prevalence of specific language impairment in kindergarten children. Journal of Speech and Hearing Research.

[b148] Weismer SE, Tomblin JB, Zhang X, Buckwalter P, Chynoweth JG, Jones M (2000). Nonword repetition performance in school-age children with and without language impairment. Journal of Speech, Language and Hearing Research.

[b149] Zubrick S, Taylor C, Lawrence D, Mitrou F, Christensen D, Dalby R (2009). The development of human capability across the lifecourse: perspectives from childhood. Australian Journal of Epidemiology.
